# Health-related quality of life in women with normogonadotropic ovulatory dysfunction: a prospective cohort study

**DOI:** 10.3389/fgwh.2026.1806970

**Published:** 2026-08-12

**Authors:** Iwona Gawron, Justyna Brodowicz, Rafal Baran, Aleksandra Bromboszcz, Marzena Wielgus, Robert Jach

**Affiliations:** 1 Faculty of Medicine, Chair of Gynecology and Obstetrics, Jagiellonian University Medical College, Krakow, Poland; 2 Clinical Department of Gynecological Endocrinology and Gynecological Oncology, University Hospital in Krakow, Krakow, Poland; 3 Faculty of Medicine, Chair of Clinical Biochemistry, Jagiellonian University Medical College, Krakow, Poland

**Keywords:** health-related quality of life, hypothalamic-pituitary-ovarian axis dysfunction, ovulatory dysfunction, polycystic ovary syndrome, SF-36

## Abstract

**Purpose:**

To compare health-related quality of life (HRQoL) in women with normogonadotropic ovulatory dysfunction, specifically polycystic ovary syndrome (PCOS) and hypothalamic-pituitary-ovarian axis dysfunction (HPOD), to that of regularly menstruating women, and to evaluate the impact of selected clinical and biochemical parameters on HRQoL.

**Methods:**

HRQoL across nine domains was compared using the RAND 36-Item Health Survey among women with regular and irregular menstruation. Correlations between clinical parameters and HRQoL scores were examined, along with correlations between biochemical parameters and HRQoL scores in anovulatory women.

**Results:**

HRQoL in the domains of Role Limitations Due to Physical Health (*p* = 0.024), Role Limitations Due to Emotional Problems (*p* < 0.001), Energy/Fatigue (*p* = 0.006), Emotional Wellbeing (*p* = 0.01), Social Functioning (*p* = 0.007), General Health (*p* < 0.001), and Health Change (*p* = 0.005) was significantly better in regularly menstruating women. No significant differences were observed in HRQoL between women with PCOS and those with HPOD (all *p* > 0.05). Women with PCOS phenotype D had a better Physical Functioning score compared to those with PCOS A and B (*p* = 0.017). Significant negative correlations were observed between Ferriman-Gallwey and Ludwig scale scores and HRQoL aspects in the entire cohort, and between dysmenorrhea, heavy menstrual bleeding, BMI, CRP concentrations, dyslipidemia, and insulin resistance and HRQoL in anovulatory women. Testosterone concentrations did not correlate with HRQoL.

**Conclusion:**

HRQoL in women with ovulatory dysfunction was worse than that of women with regular ovulation in most HRQoL domains, with no differences between PCOS and HPOD. Significant correlations were identified between clinical and biochemical health indicators and the assessed HRQoL domains.

## 1 Introduction

Normogonadotropic ovulatory dysfunction is primarily associated with polycystic ovary syndrome (PCOS), which affects approximately 10%–13% of women of reproductive age (1) and accounts for 60%–91% of anovulation cases in Group 2 of the World Health Organization (WHO) classification, depending on the diagnostic criteria used (2–4). The remaining cases of unexplained anovulation that fall within this group, not meeting the criteria for PCOS, may be attributed to hypothalamic-pituitary-ovarian axis dysfunction (HPOD) (2, 3). PCOS is a heterogeneous condition with a complex and unresolved etiology, defined by a combination of features such as oligo-anovulation, hyperandrogenism, and polycystic ovarian morphology (PCOM), in accordance with the Rotterdam criteria (1). In addition to these defining characteristics, PCOS is associated with metabolic, endocrine, and psychological disturbances that impact overall health. The latest analysis revealed a global prevalence of PCOS, diagnosed according to Rotterdam criteria, at 12.1%, with the highest rates of 15.1% occurring in the eastern Mediterranean, followed by 14.3% in Southeast Asia, 11.7% in Europe, 10.5% in the Americas, and 9.1% in the Western Pacific region, with no data available from Africa, indicating likely variations in health burdens associated with this condition across different global regions (5). While PCOS has been extensively studied, the prevalence, mechanisms of disrupted ovulation, and associated health complications in HPOD have not received adequate attention, resulting in HPOD being a less understood aspect of ovarian disorders (6).

Ovulatory dysfunctions, manifesting as irregular menstrual cycles (7, 8), have been linked to various systemic conditions, including metabolic syndrome, diabetes, autoimmune diseases, and specific pregnancy complications. Consequently, irregular ovulation may serve as an indicator of an individual's overall health status (9). Difficulties in planning daily activities and concerns about unintended pregnancy or anovulatory infertility are linked to workplace challenges, familial stress, compromised sexual function, a lack of control over one's body, and reduced self-esteem (10), all contributing to increased anxiety and depressive symptoms (11). Additionally, visible secondary symptoms associated with insulin resistance and hyperandrogenism, such as obesity, hirsutism, androgenic alopecia, and severe acne, along with dysmenorrhea and premenstrual syndrome accompanying menstrual disorders, can further exacerbate psychological distress (11, 12). The cumulative impact of these factors reduces the health-related quality of life (HRQoL), defined as a multifaceted construct influenced by satisfaction in the physical, emotional, social, and economic dimensions of an individual's life (13). As there is currently no effective cure for normogonadotropic ovulatory dysfunctions, treatment is limited to managing symptoms, with the approach depending on the specific therapeutic goals. For women not seeking conception, this involves pharmacological regulation of menstrual cycles, while those pursuing pregnancy may require medically assisted reproductive technologies. Although lifestyle modifications may improve body weight and hyperandrogenism in women with PCOS, their effect on glucose tolerance is uncertain, and evidence regarding menstrual regularity, obstetric outcomes and HRQoL is lacking (14–16).

Given the variable efficacy of therapeutic interventions and the chronicity of these conditions, along with their potential effects on emotional and social wellbeing, there has been a growing interest in researching the impact of ovulatory disorders on HRQoL. Furthermore, ovulatory dysfunctions, particularly PCOS, significantly affect the healthcare system. A recent meta-analysis has demonstrated that the economic burden associated with the obstetric outcomes and chronic health complications related to PCOS, along with the costs of initial diagnosis and treatment for irregular menstruation, hirsutism, and infertility, amounts to $8 billion annually in the United States (17). A comprehensive analysis based on the Global Burden of Disease 2021 data revealed that the global burden of PCOS grew from 1990 to 2021, including a 47-fold increase in the number of new cases, a 28.05% rise in the global age-standardized prevalence rate, a 27.93% increase in age-standardized incidence rates, and a 27.48% rise in the age-standardized rate of disability-adjusted life years (18). These data suggest that greater vigilance among clinicians, increased public awareness and evidence-informed patient resources are needed, necessitating enhanced investment in research and education to improve the diagnosis of PCOS, which would alleviate the effects of this syndrome on the economy, health, and quality of life (17).

The recommendations on health and fertility in women with normogonadotropic ovulatory dysfunction (6) have not thoroughly addressed HRQoL aspects, merely suggesting their assessment without differentiating between PCOS phenotypes or considering women with HPOD, as they have equated Group 2 to PCOS, potentially overlooking those who face unrecognized negative health consequences associated with this condition. Evaluating the associated physical, psychological, and social impairments is essential for developing effective prevention and treatment strategies to address health issues related to ovulatory dysfunction. The primary aim of the study was to assess and compare the HRQoL of women with ovulatory disorders, focusing on PCOS and HPOD, to that of regularly menstruating women, utilizing the RAND 36-Item Health Survey (version 1.0) (19). The secondary aim was to evaluate the relationship between selected clinical and biochemical parameters and HRQoL. It was hypothesized that women with PCOS and HPOD would report poorer HRQoL than regularly menstruating women, with women with PCOS exhibiting poorer HRQoL than those with other forms of ovulatory dysfunction. In addition, it was hypothesized that clinical and biochemical characteristics would be associated with HRQoL within each group.

## 2 Methods

A prospective cohort study was conducted from July 1, 2023, to December 30, 2024, following the approval of the Jagiellonian University Bioethics Committee (reference number 118.6120.21.2023) and registration with ClinicalTrials.gov (ID: NCT06208995). Participants were recruited consecutively as they presented for evaluation of ovulatory disorders at the Clinical Department of Gynecological Endocrinology and Gynecological Oncology, University Hospital in Krakow, and met the study inclusion and exclusion criteria. Women in the control group were recruited consecutively during routine outpatient gynecological visits and invited to participate after confirmation that they met the study inclusion and exclusion criteria. The research adhered to the Declaration of Helsinki, with all participants providing informed written consent prior to participation. The study included participants aged 18–45, excluding those who had undergone previous ovarian surgery. Women were included in the study group of irregular menstrual cycles if their cycle lengths were less than 21 days or greater than 35 days. The women underwent a standard gynecological assessment, which included pelvic ultrasound conducted through transvaginal and/or transabdominal approaches, utilizing EV2-10A volume endocavity and CV1-8A convex transducers with the Samsung HERA W10 Elite device (Suwon, South Korea). Diagnosis of polycystic ovarian morphology (PCOM) required at least 20 follicles measuring 2–9 mm and/or an ovarian volume of at least 10 mL, excluding the presence of a corpus luteum, dominant follicle, or functional lesion (1). Excess body hair was assessed with the modified Ferriman-Gallwey scale (mFG) (20), while androgenic alopecia was evaluated using the Ludwig scale (LS) (21). Women rated menstrual pain severity on a scale of 1–10, with dysmenorrhea defined as a pain intensity above five. HRQoL was evaluated using the self-administered 36-item RAND Health Survey (version 1.0), examining eight dimensions of health – Physical functioning, Role limitations due to physical health, Role limitations due to emotional problems, Energy/fatigue, Emotional wellbeing, Social functioning, Pain, and General health – and perceived Change in Health status (19), with scores calculated according to the guidelines provided at https://www.rand.org/health-care/surveys_tools/mos/36-item-short-form/scoring.html. The SF-36 is a validated generic questionnaire widely used in research and clinical practice to assess an individual's physical and mental health, serving as one of the most frequently employed instruments for measuring HRQoL in women with PCOS (22).

Assuming a confidence level of 95%, a margin of error of 5%, and a population proportion of 50%, along with an estimated annual number of 1,100 women undergoing diagnosis for menstrual irregularities, a minimum sample size of 312 participants was required. The healthy control group, consisting of women undergoing routine gynecological examinations at a rate of approximately 300 annually, required a minimum sample size of 208 participants. To enhance the sample size, women meeting the study criteria were consecutively included as they presented at the center, with only those who did not consent to participate or who failed to return a completed questionnaire not being enrolled, as only fully completed surveys were included in the analysis.

### 2.1 Biochemical analysis

Women with irregular menstrual cycles underwent biochemical blood tests using a 10 mL fasting venous sample analyzed with the Cobas PRO/e801 system (Roche Diagnostics, Basel, Switzerland). Serum concentrations of follicle-stimulating hormone (FSH), luteinizing hormone (LH), prolactin (PRL), Anti-Müllerian hormone (AMH), estradiol, testosterone, sex hormone binding globulin (SHBG), dehydroepiandrosterone sulfate (DHEA-S), fasting insulin (FI) and at 120 min of the 75 g-glucose tolerance test (120'75OGTTI), thyroid-stimulating hormone (TSH), free triiodothyronine (fT3), free thyroxine (fT4), thyroid peroxidase (TPOAb) and thyroglobulin antibodies (TGAb) were measured utilizing electrochemiluminescence immunoassay (ECLIA). Fasting glucose (FG) and at 120 min of 75OGTT (120'75OGTTG), triglycerides (TG), and total cholesterol (TC) were assessed using enzymatic methods, while high-density lipoprotein cholesterol (HDL) was evaluated spectrophotometrically. C-reactive protein (CRP) was analyzed using an immunoturbidimetric method. Low-density lipoprotein (LDL) cholesterol was calculated using the Friedewald equation (23). Homeostasis model assessment for insulin resistance (HOMA-IR) was calculated by multiplying FI by FG and dividing by 22.5. The free androgen index (FAI) was obtained by dividing total testosterone concentration by SHBG concentration. The upper reference range for prolactin concentration was 496 *μ*IU/mL, and FSH concentration was 12.5 mIU/mL during the follicular phase. Hyperandrogenemia was recognized as a serum testosterone concentration exceeding 1.67 nmol/L.

### 2.2 Diagnosis of ovulatory dysfunctions

PCOS was diagnosed based on the Rotterdam criteria (1), with phenotyping as follows: A - oligoovulation, hyperandrogenism (hyperandrogenemia or FAI > 5), and PCOM; B - oligoovulation and hyperandrogenism; C – regular ovulation, hyperandrogenism and PCOM, D - oligoovulation and PCOM without hyperandrogenism. Phenotype C was not represented because an irregular menstrual cycle was an inclusion criterion. Normogonadotropic ovulatory dysfunction not meeting PCOS criteria was classified as HPOD. Among these women, those who had a higher concentration of FSH, yet did not meet the criteria for the diagnosis of premature ovarian insufficiency (24), as well as those with a non-pathological increase in prolactin concentration (25) equivalent to idiopathic hyperprolactinemia, were identified for the purpose of further calculations.

### 2.3 Statistical analysis

The analysis of quantitative variables was conducted using descriptive statistics, including mean, standard deviation, median, quartiles, and the minimum and maximum values. Qualitative variables were analyzed by determining the absolute and percentage frequencies of all possible values. The distribution of variables was tested for normality using the Kolmogorov–Smirnov test. The Mann–Whitney test was employed to compare quantitative variables between two groups, while the Kruskal–Wallis test was used for comparisons across three or more groups. In cases of statistically significant differences, Dunn's *post-hoc* test was applied. Univariate and multiple linear regression analyses were performed to identify potential predictors of the quantitative outcome, with results presented as regression parameters along with 95% confidence intervals. A significance level of 0.05 was adopted, with *p*-values below 0.05 interpreted as indicative of significant relationships. The analysis was conducted using R, version 4.5.1 (26).

## 3 Results

**Figure 1:**
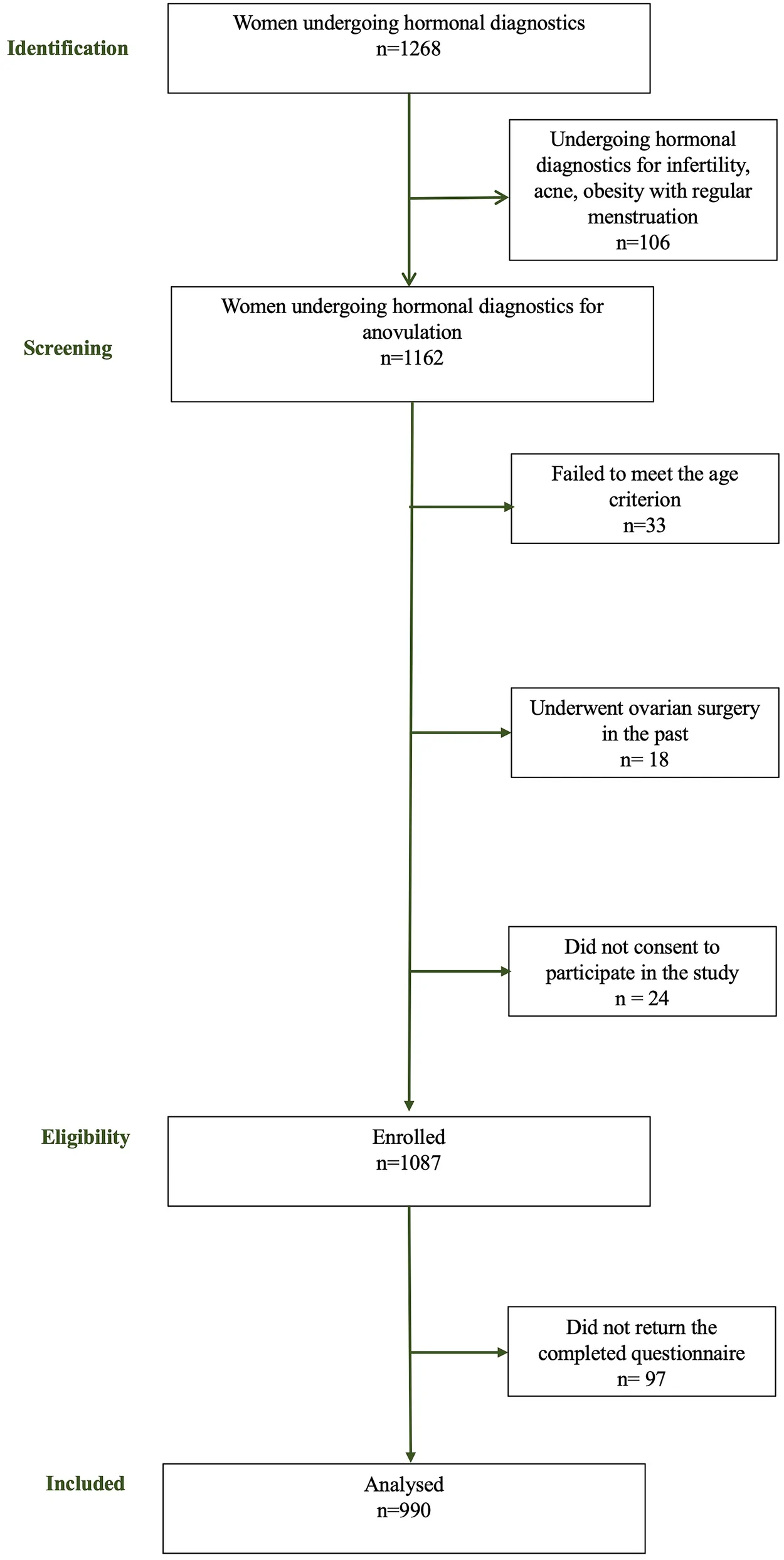
Recruitment flow diagram.

The recruitment of participants was presented in the flow diagram (Figure 1). The study group comprised 990 consecutively enrolled women with irregular menstruation, while the control group included 335 consecutively assessed women with regular ovulation. The database has been made publicly available at https://doi.org/10.7910/DVN/IJSPZH. The comparative characteristics of the studied women with respect to selected clinical variables were presented in Table 1. Women with regular cycles were significantly older than those with irregular cycles (*p* < 0.001), while the mFG score was significantly higher in women with irregular cycles (*p* = 0.032). The detailed clinical and biochemical characterization of the subpopulation of women with irregular menstruation was shown in Supplementary Table 1.

**Table 1:** Comparative characteristics of the studied population based on selected clinical parameters.

Variable		Irregular cycles (*N* = 990)	Regular cycles (*N* = 335)	Total (*N* = 1,325)	p
Age [years]	Mean (SD)	26.07 (5.54)	29.32 (6.07)	26.89 (5.85)	*p* < 0.001* r = 0.242
	Median (quartiles)	25 (22,29)	29 (25,34)	26 (22,31)	
	Range	18–50	18–45	18–50	
mFG [n]	Mean (SD)	5.78 (4.7)	5.11 (4.12)	5.61 (4.57)	*p* = 0.032* r = 0.059
	Median (quartiles)	5 (2,8)	4 (2,8)	5 (2,8)	
	Range	0–32	0–18	0–32	
LS	0	3 (0.30%)	0 (0.00%)	3 (0.23%)	*p* = 0.41
	I-1	624 (63.03%)	208 (62.09%)	832 (62.79%)	
	I-2	269 (27.17%)	97 (28.96%)	366 (27.62%)	
	I-3	77 (7.78%)	21 (6.27%)	98 (7.40%)	
	I-4	14 (1.41%)	5 (1.49%)	19 (1.43%)	
	II-1	2 (0.20%)	3 (0.90%)	5 (0.38%)	
	II-2	1 (0.10%)	1 (0.30%)	2 (0.15%)	
Weight [kg]	Mean (SD)	68.29 (18.14)	66.76 (14.39)	67.9 (17.28)	*p* = 0.87 r = 0.004
	Median (quartiles)	63.5 (55,77)	63 (57,75)	63 (55,76)	
	Range	40–169	43–115	40–169	
Height [m]	Mean (SD)	1.66 (0.06)	1.66 (0.06)	1.66 (0.06)	*p* = 0.425 r = 0.022
	Median (quartiles)	1.65 (1.62,1.7)	1.66 (1.62,1.7)	1.65 (1.62,1.7)	
	Range	1.49–1.88	1.5–1.8	1.49–1.88	
BMI [kg/m²]	Mean (SD)	24.77 (6.21)	24.25 (5.39)	24.64 (6.02)	*p* = 0.598 r = 0.014
	Median (quartiles)	23.02 (20.2,27.77)	22.99 (20.38,26.7)	23.01 (20.2,27.48)	
	Range	14.53–50.19	16.37–48	14.53–50.19	
BMI classification	Thinness	14 (1.41%)	2 (0.60%)	16 (1.21%)	*p* = 0.2
	Underweight	75 (7.58%)	23 (6.87%)	98 (7.40%)	
	Normal weight	537 (54.24%)	203 (60.60%)	740 (55.85%)	
	Overweight	187 (18.89%)	57 (17.01%)	244 (18.42%)	
	Obesity	91 (9.19%)	33 (9.85%)	124 (9.36%)	
	Obesity, class II	58 (5.86%)	13 (3.88%)	71 (5.36%)	
	Obesity, class III	28 (2.83%)	4 (1.19%)	32 (2.42%)	
Dysmenorrhea	Yes	468 (47.27%)	163 (48.66%)	631 (47.62%)	*p* = 0.704
	No	522 (52.73%)	172 (51.34%)	694 (52.38%)	
Abnormal uterine bleeding	IMB	77 (7.78%)	15 (4.48%)	92 (6.95%)	*p* = 0.038*
	HMB	298 (30.10%)	119 (35.52%)	417 (31.47%)	
	No	615 (62.12%)	201 (60%)	816 (61.58%)	

*p* - Qualitative variables: chi-squared or Fisher's exact test, Quantitative variables: Mann–Whitney test, *statistically significant (*p* < 0.05), SD - standard deviation, quartiles: Q1(lower quartile), Q3 (upper quartile), mFG – modified Ferriman-Gallwey scale score, LS – Ludwig scale score, BMI – body mass index; Effect size (r - for comparing two groups): <0.01: negligible effect; 0.01 to <0.06: Small effect; 0.06 to <0.14: Moderate effect; ≥ 0.14: Large effect.

### 3.1 Health-related quality of life (HRQoL) in women with irregular menstrual cycles compared to those with regular menstrual cycles

HRQoL measure scores across various dimensions were significantly higher in women with regular menstrual cycles compared to those with irregular cycles, as delineated in Table 2. Specifically, these included the Role Limitations due to Physical Health (*p* = 0.024), Role Limitations due to Emotional Problems (*p* < 0.001), Energy/Fatigue (*p* = 0.006), Emotional Wellbeing (*p* = 0.01), Social Functioning (*p* = 0.007), General Health (*p* < 0.001), and Health Change (*p* = 0.005) measures.

**Table 2:** Comparison of health-related quality of life (HRQoL) domains according to the SF-36 questionnaire among women with irregular and regular menstrual cycles.

Quality of life domain	Group	N	Mean (SD)	Median (quartiles)	Range	p
Physical Functioning	Irregular cycles	990	90.57 (12.9)	95 (85,100)	15–100	*p* = 0.069 r = 0.048
	Regular cycles	335	91.82 (12.2)	95 (90,100)	25–100	
	Total	1,325	90.88 (12.73)	95 (85,100)	15–100	
Role Limitations due to Physical Health	Irregular cycles	990	70.95 (32.93)	75 (50,100)	0–100	*p* = 0.024* r = 0.059
	Regular cycles	335	73.21 (35.57)	100 (50,100)	0–100	
	Total	1,325	71.52 (33.62)	75 (50,100)	0–100	
Role Limitations due to Emotional Problems	Irregular cycles	990	62.56 (38.96)	66.7 (33.3, 100)	0–100	*p* < 0.001* r = 0.091
	Regular cycles	335	70.75 (38.61)	100 (33.3,100)	0–100	
	Total	1,325	64.63 (39.02)	66.7 (33.3,100)	0–100	
Energy/Fatigue	Irregular cycles	990	46.91 (17.08)	45 (35,60)	0–100	*p* = 0.006* r = 0.075
	Regular cycles	335	50.24 (17.91)	50 (40,65)	10–100	
	Total	1,325	47.75 (17.35)	50 (35,60)	0–100	
Emotional Well-Being	Irregular cycles	990	58.91 (16.17)	60 (48,72)	4–100	*p* = 0.01* r = 0.071
	Regular cycles	335	61.3 (17.61)	64 (48,76)	8–100	
	Total	1,325	59.51 (16.57)	60 (48,72)	4–100	
Social Functioning	Irregular cycles	990	64.04 (24.72)	62.5 (50,87.5)	0–100	*p* = 0.007* r = 0.074
	Regular cycles	335	67.94 (24.74)	75 (50,87.5)	0–100	
	Total	1,325	65.03 (24.78)	62.5 (50,87.5)	0–100	
Pain	Irregular cycles	990	64.94 (23.17)	67.5 (55,80)	0–100	*p* = 0.115 r = 0.043
	Regular cycles	335	67.4 (21.79)	67.5 (55, 80)	10–100	
	Total	1,325	65.56 (22.85)	67.5 (55, 80)	0–100	
General Health	Irregular cycles	990	45.91 (12.58)	45 (35,55)	10–85	*p* < 0.001* r = 0.129
	Regular cycles	335	49.45 (12.25)	50 (40,55)	10–80	
	Total	1,325	46.8 (12.59)	45 (40,55)	10–85	
Health Change	Irregular cycles	990	46.98 (20.86)	50 (25,50)	0–100	*p* = 0.005* r = 0.071
	Regular cycles	335	50.82 (20.26)	50 (50,50)	0–100	
	Total	1,325	47.95 (20.77)	50 (25,50)	0–100	

*p* - Mann–Whitney test, * statistically significant (*p* < 0.05), SD - standard deviation, quartiles: Q1(lower quartile), Q3 (upper quartile), Range: minimum value - maximum value; Effect size (r - for comparing two groups): <0.01: negligible effect; 0.01 to <0.06: Small effect; 0.06 to <0.14: Moderate effect; ≥ 0.14: Large effect.

### 3.2 Comparative analysis of health-related quality of life (HRQoL) in women with distinct conditions of ovulatory dysfunction

No significant differences were observed in HRQoL aspects between women with PCOS and those with HPOD, whether considered collectively or with a distinction made for women with tendencies toward higher concentrations of FSH or prolactin, as shown in Table 3.

**Table 3:** Comparison of health-related quality of life (HRQoL) aspects according to the SF-36 questionnaire among women with irregular menstrual cycles across four distinct anovulatory groups - polycystic ovary syndrome (PCOS), idiopathic hyperprolactinemia, hypergonadotropic anovulation, and hypothalamic-pituitary-ovarian axis dysfunction (HPOD).

Health-related quality of life domain	Ovulatory dysfunction	N	Mean (SD)	Median (quartiles)	Range	p
Physical functioning	PCOS	667	90.53 (12.95)	95 (85,100)	15–100	*p* = 0.706 r = 0.012
	No PCOS	323	90.63 (12.8)	95 (90,100)	40–100	
	PCOS	667	90.53 (12.95)	95 (85,100)	15–100	*p* = 0.285 *η*²=0.001
	Idiopathic hyperprolactinemia	93	90.59 (12.85)	95 (90,100)	40–100	
	Inconclusive FSH elevation	18	85.83 (15.46)	92.5 (76.25,95)	50–100	
	HPOD	212	91.06 (12.51)	95 (85,100)	45–100	
Role limitations due to physical health	PCOS	667	71.14 (32.72)	75 (50,100)	0–100	*p* = 0.872 r = 0.005
	No PCOS	323	70.57 (33.39)	75 (50,100)	0–100	
	PCOS	667	71.14 (32.72)	75 (50,100)	0–100	*p* = 0.837 η²=−0.002
	Idiopathic hyperprolactinemia	93	68.28 (34.8)	75 (50,100)	0–100	
	Inconclusive FSH elevation	18	77.78 (26.97)	75 (75,100)	0–100	
	HPOD	212	70.97 (33.28)	75 (50,100)	0–100	
Role limitations due to emotional problems	PCOS	667	61.27 (39.15)	66.7 (33.3,100)	0–100	*p* = 0.139 r = 0.044
	No PCOS	323	65.22 (38.5)	66.7 (33.3,100)	0–100	
	PCOS	667	61.27 (39.15)	66.7 (33.3,100)	0–100	*p* = 0.236 η²=0.001
	Idiopathic hyperprolactinemia	93	65.95 (38.38)	66.7 (33.3,100)	0–100	
	Inconclusive FSH elevation	18	77.78 (34.3)	100 (66.7,100)	0–100	
	HPOD	212	63.83 (38.85)	66.7 (33.3,100)	0–100	
Energy/fatigue	PCOS	667	46.21 (17.03)	45 (35,60)	0–100	*p* = 0.064 r = 0.059
	No PCOS	323	48.36 (17.13)	50 (35,60)	0–100	
	PCOS	667	46.21 (17.03)	45 (35,60)	0–100	*p* = 0.104 η²=0.003
	Idiopathic hyperprolactinemia	93	47.96 (16.32)	45 (35,55)	15–100	
	Inconclusive FSH elevation	18	54.17 (18.01)	55 (46.25,63.75)	15–85	
	HPOD	212	48.04 (17.4)	50 (35,60)	0–80	
Emotional well-being	PCOS	667	58.56 (15.9)	60 (46,72)	16–96	*p* = 0.281 r = 0.034
	No PCOS	323	59.63 (16.72)	60 (48,72)	4–100	
	PCOS	667	58.56 (15.9)	60 (46,72)	16–96	*p* = 0.259 η²=0.001
	Idiopathic hyperprolactinemia	93	61.42 (15.93)	64 (48,76)	24–100	
	Inconclusive FSH elevation	18	64.44 (16.8)	62 (52,75)	32–96	
	HPOD	212	58.44 (16.99)	60 (48,72)	4–92	
Social functioning	PCOS	667	63.4 (24.86)	62.5 (50,87.5)	0–100	*p* = 0.297 r = 0.033
	No PCOS	323	65.36 (24.42)	62.5 (50,87.5)	0–100	
	PCOS	667	63.4 (24.86)	62.5 (50,87.5)	0–100	*p* = 0.2 η²=0.002
	Idiopathic hyperprolactinemia	93	67.74 (22.9)	62.5 (50,87.5)	25–100	
	Inconclusive FSH elevation	18	73.61 (24.21)	75 (62.5,96.88)	12.5–100	
	HPOD	212	63.62 (24.96)	62.5 (50,87.5)	0–100	
Pain	PCOS	667	65.06 (23.08)	67.5 (55,80)	0–100	*p* = 0.947 r = 0.002
	No PCOS	323	64.71 (23.4)	67.5 (55,80)	0–100	
	PCOS	667	65.06 (23.08)	67.5 (55,80)	0–100	*p* = 0.75 η²=−0.002
	Idiopathic hyperprolactinemia	93	66.64 (22.56)	67.5 (55,80)	10–100	
	Inconclusive FSH elevation	18	68.06 (23.6)	67.5 (55,80)	10–100	
	HPOD	212	63.58 (23.77)	67.5 (47.5,80)	0–100	
General health	PCOS	667	45.4 (12.42)	45 (35,55)	10–85	*p* = 0.081 r = 0.055
	No PCOS	323	46.96 (12.86)	45 (40,55)	14–85	
	PCOS	667	45.4 (12.42)	45 (35,55)	10–85	*p* = 0.255 η²=0.001
	Idiopathic hyperprolactinemia	93	46.94 (12.6)	45 (40,55)	25–85	
	Inconclusive FSH elevation	18	44.17 (10.04)	45 (36.25,50)	30–65	
	HPOD	212	47.21 (13.2)	50 (38.75,55)	14–80	
Health change	PCOS	667	46.42 (19.41)	50 (25,50)	0–100	*p* = 0.284 r = 0.031
	No PCOS	323	48.14 (23.57)	50 (25,50)	0–100	
	PCOS	667	46.42 (19.41)	50 (25,50)	0–100	*p* = 0.588 η²=−0.001
	Idiopathic hyperprolactinemia	93	46.77 (21.56)	50 (25,50)	0–100	
	Inconclusive FSH elevation	18	54.17 (30.01)	50 (31.25, 68.75)	0–100	
	HPOD	212	48.23 (23.84)	50 (25,50)	0–100	

*p* - Mann–Whitney test, SD - standard deviation, quartiles: Q1(lower quartile), Q3 (upper quartile), Range: minimum value - maximum value, PCOS - polycystic ovary syndrome, HPOD - hypothalamic-pituitary-ovarian axis dysfunction; Effect size (r - for comparing two groups, η² - for comparing more than two groups): <0.01: negligible effect; 0.01 to <0.06: Small effect; 0.06 to <0.14: Moderate effect; ≥ 0.14: Large effect.

### 3.3 Comparison of health-related quality of life (HRQoL) among women with distinct phenotypes of polycystic ovary syndrome (PCOS)

When comparing the HRQoL aspects among women with different PCOS phenotypes, a significant difference was identified exclusively in the domain of Physical Functioning, which was superior in women with PCOS D compared to those with PCOS A and B (*p* = 0.017), as evidenced in Table 4.

**Table 4:** Comparison of health-related quality of life (HRQoL) aspects according to the SF-36 questionnaire among women with different phenotypes of polycystic ovary syndrome (PCOS).

Health-related quality of life domain	PCOS phenotype	N	Mean (SD)	Median (quartiles)	Range	p
Physical Functioning	PCOS A	331	90.23 (13)	95 (85,100)	25–100	*p* = 0.017* η²=0.009 PCOS D > PCOS A,B
	PCOS B	153	88.59 (14.69)	95 (80,100)	15–100	
	PCOS D	183	92.71 (10.9)	95 (90,100)	40–100	
Role limitations due to Physical Health	PCOS A	331	71.9 (31.38)	75 (50,100)	0–100	*p* = 0.939 η²=−0.003
	PCOS B	153	70.42 (35.06)	75 (50,100)	0–100	
	PCOS D	183	70.36 (33.24)	75 (50,100)	0–100	
Role limitations due to Emotional Problems	PCOS A	331	61.43 (38.75)	66.7 (33.3,100)	0–100	*p* = 0.996 η²=−0.003
	PCOS B	153	60.78 (40.67)	66.7 (33.3,100)	0–100	
	PCOS D	183	61.38 (38.79)	66.7 (33.3,100)	0–100	
Energy/Fatigue	PCOS A	331	46.18 (17.34)	45 (35,60)	0–100	*p* = 0.982 η²=−0.003
	PCOS B	153	45.98 (16.98)	45 (35,60)	10–80	
	PCOS D	183	46.45 (16.59)	45 (35,60)	0–90	
Emotional Well-Being	PCOS A	331	58.46 (15.95)	60 (44,72)	20–96	*p* = 0.602 η²=−0.001
	PCOS B	153	57.46 (16.5)	60 (44,72)	16–88	
	PCOS D	183	59.65 (15.29)	64 (48,70)	20–92	
Social Functioning	PCOS A	331	62.61 (24.86)	62.5 (50,75)	0–100	*p* = 0.689 η²=−0.002
	PCOS B	153	63.73 (25.8)	62.5 (50,87.5)	0–100	
	PCOS D	183	64.55 (24.15)	62.5 (50,87.5)	12.5–100	
Pain	PCOS A	331	65.47 (23.25)	67.5 (55,80)	0–100	*p* = 0.706 η²=−0.002
	PCOS B	153	63.71 (23.36)	67.5 (47.5,80)	0–100	
	PCOS D	183	65.44 (22.59)	67.5 (55,80)	0–100	
General Health	PCOS A	331	45.29 (12.87)	45 (35,55)	10–75	*p* = 0.673 η²=−0.002
	PCOS B	153	44.9 (12.28)	45 (35,55)	20–85	
	PCOS D	183	46.01 (11.72)	45 (35,55)	20–75	
Health Change	PCOS A	331	46.18 (19.1)	50 (25,50)	0–100	*p* = 0.507 η²=−0.001
	PCOS B	153	45.42 (17.78)	50 (25,50)	0–100	
	PCOS D	183	47.68 (21.24)	50 (25,50)	0–100	

*p* - Kruskal–Wallis test + post-hoc analysis (Dunn test), SD - standard deviation, quartiles: Q1 (lower quartile), Q3 (upper quartile), Range: minimum value - maximum value, PCOS - polycystic ovary syndrome, *statistically significant (*p* < 0.05); Effect size (η² - for comparing more than two groups): <0.01: negligible effect; 0.01 to <0.06: Small effect; 0.06 to <0.14: Moderate effect; ≥ 0.14: Large effect.

### 3.4 Impact of clinical and biochemical variables on physical functioning

The impact of clinical and biochemical variables on the assessed domain of HRQoL in women with irregular menstruation was subsequently examined using both univariate (separate for each of the considered variables) and multivariate linear regression models. In examining the influence of these variables on the domain of Physical Functioning in a univariate model, a significant negative effect of dysmenorrhea (*p* < 0.001) and heavy menstrual bleeding (HMB) (*p* < 0.001) was identified, along with older age (*p* < 0.001), elevated mFG (*p* < 0.001), FAI (*p* < 0.001), HOMA-IR (*p* < 0.001), and LS scores (all *p* ≤ 0.001), and BMI (*p* < 0.001), as well as increasing concentrations of FG (*p* < 0.001), 120'75OGTTG (*p* < 0.001), FI (*p* < 0.001), 120'75OGTTI (*p* < 0.001), LDL (*p* = 0.004), TG (*p* < 0.001), and CRP (*p* < 0.001), as evidenced in Supplementary Table 2. Conversely, an increase in the concentrations of AMH (*p* = 0.005), SHBG (*p* < 0.001), and HDL (*p* < 0.001) was associated with a more favorable outcome in this area. In the multivariate analysis, dysmenorrhea (*p* = 0.001), as well as increasing age (*p* = 0.007), mFG (*p* = 0.004) and LS scores (all *p* ≤ 0.004), BMI (*p* = 0.004), and elevated concentrations of TG (*p* = 0.014) and CRP (*p* < 0.001), negatively affected the outcome of Physical Functioning, whereas increasing AMH (*p* = 0.021) concentrations had a positive effect.

### 3.5 Impact of clinical and biochemical variables on role limitations due to physical health

In the univariate analysis of Role Limitations Due to Physical Health, it was found that this outcome deteriorated with dysmenorrhea (*p* < 0.001), HMB (*p* < 0.001), the presence of a dominant follicle (*p* = 0.048), and increasing age (*p* = 0.001), mFG (*p* < 0.001), LS (all *p* ≤ 0.002), and HOMA-IR (*p* = 0.019) scores, BMI (*p* = 0.001), as well as with elevated concentrations of FI (*p* = 0.008), 120'75OGTTI (*p* = 0.021), TG (*p* = 0.002), and CRP (*p* = 0.004), as shown in Supplementary Table 3. Conversely, increasing concentration of HDL (*p* = 0.01) was associated with an improvement in this outcome. In the multivariate analysis, dysmenorrhea (*p* < 0.001), as well as increasing age (*p* = 0.032), mFG (*p* = 0.013) and LS (all *p* ≤ 0.046) scores, and elevated concentrations of CRP (*p* = 0.04), negatively affected the outcome.

### 3.6 Impact of clinical and biochemical variables on role limitations due to emotional problems

The univariate analysis of Role Limitations Due to Emotional Problems revealed a negative impact from dysmenorrhea (*p* = 0.006) and HMB (*p* = 0.012), increasing mFG (*p* = 0.003), BMI (*p* = 0.005), as well as rising CRP (*p* = 0.003) concentration, as depicted in Supplementary Table 4. The multivariate model indicated that while an increasing mFG (*p* = 0.032) score and CRP concentrations (*p* = 0.035) adversely affected outcomes related to Role Limitations Due to Emotional Problems, infertility (*p* = 0.034) exhibited a positive effect.

### 3.7 Impact of clinical and biochemical variables on energy/fatigue

In the univariate model, the Energy/Fatigue aspect exhibited poorer outcomes with dysmenorrhea (*p* < 0.001), HMB (*p* < 0.001), elevated mFG (*p* < 0.001) and LS (*p* = 0.003) scores, BMI (*p* = 0.002), and CRP (*p* = 0.036) concentrations, but improved with longer cycle lengths (*p* = 0.019) and higher HDL (*p* = 0.001) concentrations, as delineated in Supplementary Table 5. In the multivariate analysis, the influence of the presence of dysmenorrhea (*p* = 0.014) and HMB (*p* = 0.005), as well as increasing mFG (*p* = 0.002) and LS scores (*p* = 0.019 for I-2, *p* = 0.015 for I-3), remained significant.

### 3.8 Impact of clinical and biochemical variables on emotional wellbeing

In analyzing Emotional Wellbeing, the univariate model indicated that dysmenorrhea (*p* = 0.006), HMB (*p* = 0.003), higher mFG (*p* < 0.001) and LS (*p* = 0.004 for I-3) scores, BMI (*p* < 0.001), and increasing concentrations of FI (*p* = 0.038) and CRP (*p* = 0.001) negatively impacted this aspect, as presented in Supplementary Table 6. In the multivariate model, the influences of dysmenorrhea (*p* = 0.047), mFG (*p* = 0.002) and LS (*p* = 0.05 for I-3) scores remained significant, while additionally, increasing FG concentrations (*p* = 0.035) had positively affected the outcome of this HRQoL aspect.

### 3.9 Impact of clinical and biochemical variables on social functioning

The univariate analysis revealed that dysmenorrhea (*p* < 0.001), HMB (*p* < 0.001), intermenstrual bleeding (IMB) (*p* = 0.013), increasing mFG (*p* < 0.001) and LS (all *p* ≤ 0.005) scores, BMI (*p* = 0.015), and rising CRP (*p* = 0.002) concentrations negatively impacted Social Functioning outcomes, as outlined in Supplementary Table 7. In the multivariate analysis, the significant influences of dysmenorrhea (*p* = 0.041), HMB (*p* = 0.018), mFG (*p* = 0.001) and LS (all *p* ≤ 0.042) scores, as well as CRP (*p* = 0.034) concentration, were retained.

### 3.10 Impact of clinical and biochemical variables on pain

When examining the aspect of Pain, the univariate model indicated a negative impact from dysmenorrhea (*p* < 0.001) and HMB (*p* < 0.001), as well as from increasing mFG (*p* = 0.022) and LS (*p* = 0.03 for I-3) scores, while demonstrating a positive effect of increased cycle length (*p* = 0.02) and concentrations of AMH (*p* = 0.039) and HDL (*p* = 0.024), as shown in Supplementary Table 8. In the multivariate analysis, the significant influences of dysmenorrhea (*p* < 0.001) and HMB (*p* = 0.009) were maintained.

### 3.11 Impact of clinical and biochemical variables on general health

The univariate analysis showed that dysmenorrhea (*p* = 0.042) and HMB (*p* < 0.001), along with increasing age (*p* = 0.019), rising mFG (*p* < 0.001), LS (*p* ≤ 0.001 for I-2 and I-3), and HOMA-IR (*p* < 0.001) scores, BMI (*p* < 0.001), as well as elevated concentrations of FI (*p* < 0.001), 120'75OGTTI (*p* = 0.007), TG (*p* = 0.001), and CRP (*p* < 0.001), adversely affected the aspect of General Health, while increasing SHBG (*p* = 0.036) and HDL (*p* = 0.001) concentrations positively influenced it, as summarized in Supplementary Table 9. The multivariate analysis revealed a significant impact of HMB (*p* = 0.016), mFG (*p* = 0.002), and LS (*p* = 0.01 for I-2, *p* < 0.001 for I-3) scores.

### 3.12 Impact of clinical and biochemical variables on health change

The univariate model indicated that dysmenorrhea (*p* = 0.038), along with increasing mFG (*p* = 0.041) and LS (all *p* ≤ 0.023) scores, BMI (*p* = 0.009), and elevated TSH (*p* = 0.05) concentration, negatively affected the aspect of Health Change, as presented in Supplementary Table 10. Conversely, the lengthening cycle (*p* = 0.01) was positively influenced this domain. The multivariate model demonstrated that increasing LS scores (all *p* ≤ 0.029) and elevated TC (*p* = 0.015) concentrations had a negative impact on the aspect of Health Change, while the corpus luteum (*p* = 0.034) and rising concentrations of HDL (*p* = 0.015), LDL (*p* = 0.01), and TG (*p* = 0.032) positively influenced it.

### 3.13 Impact of clinical and biochemical variables on health-related quality of life (HRQoL) in regularly menstruating women

The same methodology was used to assess the impact of selected variables on HRQoL in women with regular menstrual cycles, as presented in Supplementary Table 1^1^. With respect to Physical Functioning, both univariate and multivariate models demonstrated that increasing LS (*p* = 0.036 for I-3, *p* = 0.005 for I-4, II-1, II-2 and *p* = 0.011 for I-4, II-1, II-2, respectively) scores and BMI (*p* = 0.001 and *p* = 0.004, respectively) had a negative impact on outcomes. In the domain of Role Limitations due to Physical Health, older age (*p* = 0.05) was associated with poorer outcomes in the univariate model, although no significant variables were identified in the multivariate model. Both models for Role Limitations due to Emotional Problems indicated that older age (*p* = 0.002 and *p* = 0.004, respectively), along with increasing mFG (*p* = 0.003 and *p* = 0.014, respectively) and LS (*p* = 0.017 for I-2, *p* = 0.009 for I-4, II-1, II-2 and *p* = 0.005 for I-4, II-1, II-2, respectively) scores, adversely affected the results. Similarly, older age (*p* = 0.004 and *p* = 0.009, respectively) and rising mFG (*p* = 0.003 and *p* = 0.01, respectively) scores were found to negatively impact the aspect of Energy/Fatigue in both models. The Emotional Wellbeing aspect also revealed a significant effect of LS scores in both models (*p* = 0.004 for I-4, II-1, II-2). In the Social Functioning domain, no significant effect of age was noted, while increasing mFG (*p* = 0.002 and *p* = 0.004, respectively) and LS (*p* = 0.04 for I-4, II-1, II-2 and *p* = 0.039 I-4, II-1, II-2, respectively) scores exhibited comparable negative effects in both models. None of the examined variables significantly affected the aspect of Pain. Regarding General Health, the univariate model identified negative impacts from increasing mFG (*p* < 0.001) and LS (*p* = 0.016 for I-2 and I-3) scores, as well as BMI (*p* = 0.004), all of which were significant in the multivariate model (*p* = 0.003 for mFG, *p* = 0.026 for LS I-2, *p* = 0.035 for LS I-3, *p* = 0.012 for BMI). Lastly, both models indicated solely a negative influence of LS (*p* = 0.004 for I-2 in both models) on the aspect of Health Change.

## 4 Discussion

Irregular menstruation is a relatively common condition affecting up to 25% of women, and although it can negatively impact professional performance, family life, and overall HRQoL, it is often not perceived by them as a significant health concern. Nevertheless, long-term observations reported in the literature have indicated a correlation with various health complications, including type 2 diabetes mellitus, metabolic syndrome, cardiovascular diseases, osteoporosis, and hypertensive disorders associated with pregnancy, along with an increased risk of unfavorable obstetric and neonatal outcomes, all of which may further diminish HRQoL (9, 27). The study revealed that HRQoL was significantly poorer among women with ovulatory dysfunction compared to those with regular menstruation, specifically in the domains of Role Limitations due to Physical Health, Role Limitations due to Emotional Problems, Energy/Fatigue, Emotional Wellbeing, Social Functioning, General Health, and Health Change. Only in the Physical Functioning and Pain domains were no significant differences observed. While prior research has indicated that women with menstrual irregularities may more frequently experience reduced physical fitness due to HMB and consequent anemia, as well as dysmenorrhea linked to PCOM (9, 28–30), this was not supported by the evaluation of related aspects of HRQoL in our study. A potential contributing factor may have been that the majority of participants were women with prolonged rather than shortened cycles, which resulted in a lower frequency of associated symptoms. Increasing cycle length indeed correlated with a better outcome in the Pain domain in irregularly menstruating individuals. The lack of a significant difference in the assessment of Physical Functioning between irregularly and regularly menstruating women could theoretically be attributed to the older age of the latter group, as previous studies have shown that HRQoL in most aspects improved with age after 45 compared to reproductive age, with the exception of the physical domain (31). However, attributing the observed outcome to a significant yet minor age difference between the groups – likely due to burdensome symptoms accompanying irregular menstruation that prompt earlier diagnostic evaluations – remains debatable. Although advancing age significantly negatively impacted Physical Functioning in both regularly and irregularly menstruating women, Role Limitations due to Physical Health deteriorated with age among irregularly menstruating women, while they improved among those with regular menstruation. This may indicate a decline in physical condition due to the disturbances accompanying irregular menstruation, whereas regularly menstruating women who do not experience such disturbances may also demonstrate better coping mechanisms.

The poorer HRQoL in individuals with PCOS has been attributed to the clinical conditions associated with hyperandrogenism and infertility, followed by metabolic and mental comorbidities (11). However, among women with HPOD who do not exhibit these conditions, HRQoL was not significantly different from that of women with PCOS. Indeed, HRQoL did not correlate with testosterone concentration, FAI value, or PCOM, in any of the assessed aspects. A possible explanation is that both conditions shared the principal consequences of ovulatory dysfunction, including menstrual irregularities and infertility-related concerns, which may have similarly affected HRQoL. In addition, the generic RAND-36 questionnaire assessed overall HRQoL rather than PCOS-specific concerns, which vary according to symptom profile, phenotype, and cultural context. Consequently, it may have been insufficiently sensitive to detect differences related to hirsutism, acne, or excess body weight. Finally, because participants were recruited from a tertiary referral center, both groups may have had comparable symptom severity and healthcare-seeking behavior, thereby reducing the likelihood of detecting differences in generic HRQoL.

When examining women with HPOD, no association was found between HRQoL and FSH or prolactin concentrations. In prior research, HRQoL was poorer among irregularly menstruating cancer survivors with higher FSH concentrations compared to controls, even long after diagnosis, but FSH concentration was not independently correlated with HRQoL (32). While elevated prolactin concentration in women with prolactinoma has negatively affected HRQoL (33), the aspects of HRQoL in women with idiopathic hyperprolactinemia have yet to be studied. Although limited associations were identified between idiopathic hyperprolactinemia and health complications, it was not established that these complications were dependent on prolactin concentration (34).

A comparison of HRQoL aspects among irregularly ovulating women with different PCOS phenotypes revealed a significant difference in Physical Functioning, with women of phenotype D exhibiting better performance than those of phenotypes A and B. Although previous studies have shown that women with the D phenotype experienced a better HRQoL related to acne and hirsutism, with variations in the emotional component scores across studies, no differences in Physical Functioning were observed (35–37). However, the lower prevalence of obesity and metabolic complications in individuals with this phenotype could account for the relatively better physical condition (38). In fact, while in women with irregular menstrual cycles, increasing BMI negatively affected most aspects of HRQoL in univariate models, its impact in the multivariate model persisted specifically for Physical Functioning.

Numerous additional correlations between clinical and biochemical variables and the subjective assessment of the studied domains of HRQoL have been identified. Consistent with expectations (9), dysmenorrhea negatively affected HRQoL of irregularly menstruating women across all domains in univariate models, with its impact persisting in the multivariate model for Physical Functioning, Role Limitations Due to Physical Health, Energy/Fatigue, Emotional Wellbeing, Social Functioning, and Pain. Consistent with previous studies (9), women with irregular menstruation exhibited numerous negative impacts of abnormal uterine bleeding (AUB), primarily HMB, on HRQoL aspects in univariate models, which persisted in the multivariate model for the areas of Energy/Fatigue, Social Functioning, Pain, and General Health. Interestingly, no significant effect of AUB or dysmenorrhea on the examined aspects of HRQoL was found in women with regular menstruation.

Among the remaining clinical parameters, a notably large number of associations were identified between increasing mFG and LS scores, which significantly diminished HRQoL across most areas in univariate models. These associations remained significant in the domains of Physical Functioning, Role Limitations Due to Physical Health, Role Limitations Due to Emotional Problems, Energy/Fatigue, Emotional Wellbeing, Social Functioning, and General Health, suggesting a relationship between clinical hyperandrogenism and the subjective assessment of HRQoL, thereby supporting other research, particularly regarding emotional components (39). In contrast, no such associations were found for hyperandrogenemia, thereby reinforcing previous findings (40). This finding suggests that the visible clinical manifestations of hyperandrogenism have a more direct impact on women's perceptions of their health and wellbeing compared to biochemical abnormalities alone. Symptoms such as hirsutism and androgenic alopecia can profoundly affect body image, self-esteem, and social functioning, while elevated androgen concentrations that do not present with clinical symptoms tend to be largely unnoticeable to them.

Irregular ovulation associated with certain medical conditions can serve as an indicator of overall health (9), a notion that may be further supported by the significant correlations observed between biochemical indicators of metabolic status and the studied domains of HRQoL. Among the biochemical parameters, significant associations were observed in univariate models, particularly between increasing concentrations of CRP and declining HRQoL across most of the studied domains, with this effect persisting in the multivariate model in the areas of Physical Functioning, Role Limitations Due to Physical Health, Role Limitations Due to Emotional Problems, and Social Functioning, thus supporting the conclusions of earlier research (40). Elevated plasma concentrations of CRP, being an established inflammatory marker, serve as a reliable indicator of overall health status, reflecting an increased risk of or presence of chronic conditions, including dyslipidemia, diabetes, metabolic syndrome, and cardiovascular diseases (41–43), and depression (44). Numerous correlations analogous to those observed for CRP – seen between dysmenorrhea and aspects of HRQoL – additionally suggest a recently investigated relationship between CRP concentrations and chronic pain (45). Although serum AMH concentrations have been investigated as predictors of infertility treatment outcomes, including IVF (46, 47) and artificial insemination in women with PCOS (48), no correlation has been demonstrated between AMH and HRQoL domains related to mental health. In this study, higher AMH concentrations were associated with significantly better scores in physical health domains, specifically Physical Functioning and Pain. This association may reflect the relatively small proportion of participants with infertility or actively pursuing pregnancy, as well as the generally better physical functioning observed among younger women, in whom AMH concentrations are typically higher.

Despite the numerous interconnected symptoms and health consequences associated with ovulatory disorders, dissatisfaction persists among women regarding the delayed diagnosis and unsatisfactory management of PCOS and its complications (49). Considering the persistent knowledge gap regarding the experiences associated with PCOS, the most common endocrinopathy in women of reproductive age (39), there is a particularly pressing need to evaluate and enhance the experiences of women with other, less specific conditions related to disrupted ovulation. As demonstrated in the study, their HRQoL was worse than that of women with regular ovulation and was not better than that of women with PCOS. The findings of the study have important implications for clinical practice. Because impaired HRQoL was observed in both women with PCOS and those with other forms of ovulatory dysfunction, early diagnosis and comprehensive evaluation of ovulatory disorders may facilitate the timely implementation of individualized management strategies. In addition to treating the underlying endocrine disorder, routine assessment of HRQoL may help identify women who could benefit from targeted psychological support, lifestyle interventions, or symptom-directed treatment, thereby improving overall functioning and wellbeing.

A significant strength of the study is its large sample size and high response rate for completed questionnaires, which enabled the collection of reliable research findings. An important advantage is that the study focused not only on PCOS itself but also on its phenotypes. Additionally, within the HPOD arm, it identified women with elevated concentrations of FSH and prolactin, allowing for a more detailed interpretation of the results.

A limitation of the study is its single-center design and reliance on patient-reported data. The single-center design may have limited the generalizability of the findings to other populations and healthcare settings. In addition, HRQoL was assessed using a validated self-reported questionnaire and was therefore subject to reporting and response biases, although the use of a standardized instrument likely reduced their impact. The study groups were recruited from a clinical population rather than from the general community, which may have introduced selection bias by overrepresenting women with greater symptom severity and higher healthcare utilization, and this should be taken into account when interpreting the study findings. The absence of formal matching for specific clinical variables may have contributed to residual confounding.

## Data Availability

The datasets presented in this study can be found in online repositories. The names of the repository/repositories and accession number(s) can be found below: The database has been made publicly available at https://doi.org/10.7910/DVN/IJSPZH. Data collected during the study are also available from the authors upon request.
