# Supplementary material for: Health-related quality of life in women with normogonadotropic ovulatory dysfunction: a prospective cohort study
Source: Front Glob Womens Health. 2026 Aug 12;7:1806970. doi: 10.3389/fgwh.2026.1806970 (PMC13506369; doi:10.3389/fgwh.2026.1806970)
Supplement: Supplementary file 1 [file Table1.docx]

Supplementary Material

Supplementary Table 1. Characteristics of women with irregular menstrual cycles based on selected clinical and biochemical parameters.

| **Variable** | | **Total (N=990)** |
| --- | --- | --- |
| Cycle length [days] | Mean (SD) | 78.59 (81.46) |
|  | Median (quartiles) | 46 (37,78) |
|  | Range | 0-700 |
| Infertility | No | 881 (88.99%) |
|  | Yes | 109 (11.01%) |
| Dysmenorrhea | No | 522 (52.73%) |
|  | Yes | 468 (47.27%) |
| Abnormal uterine  bleeding | No | 615 (62.12%) |
|  | HMB | 298 (30.10%) |
|  | IMB | 77 (7.78%) |
| Right Ovarian Volume [ml] | Mean (SD) | 11.31 (7.11) |
|  | Median (quartiles) | 10.2 (7.01,14.01) |
|  | Range | 0.24-93.12 |
| Left Ovarian Volume [ml] | Mean (SD) | 10.6 (10.51) |
|  | Median (quartiles) | 8.9 (6,12.8) |
|  | Range | 0.4-194 |
| Polycystic ovarian morphology | No | 436 (44.04%) |
|  | Yes | 554 (55.96%) |
| Dominant follicle | No | 830 (83.84%) |
|  | Yes | 160 (16.16%) |
| Corpus Luteum | No | 893 (90.20%) |
|  | Yes | 97 (9.80%) |
| Simple Cyst | No | 951 (96.06%) |
|  | Yes | 39 (3.94%) |
| Ovarian lesion | No | 923 (93.23%) |
|  | Unilateral | 63 (6.36%) |
|  | Bilateral | 4 (0.40%) |
| Anti-Müllerian hormone [pmol/l] | Mean (SD) | 43.75 (33.72) |
|  | Median (quartiles) | 37.1 (23.02,54.6) |
|  | Range | 0-398 |
| FSH [mIU/ml] | Mean (SD) | 6.37 (7.93) |
|  | Median (quartiles) | 5.67 (4.14,6.93) |
|  | Range | 0.33-116 |
| LH [mIU/ml] | Mean (SD) | 12.92 (11.38) |
|  | Median (quartiles) | 10.3 (5.83,16.6) |
|  | Range | 0.3-134 |
| Estradiol [pmol/l] | Mean (SD) | 364.22 (372.4) |
|  | Median (quartiles) | 231.5 (155,459) |
|  | Range | 18.4-4433 |
| Prolactin [μIU/ml] | Mean (SD) | 345.72 (297.93) |
|  | Median (quartiles) | 303 (210.5,420) |
|  | Range | 30.7-6777 |
| Testosterone [nmol/l] | Mean (SD) | 1.67 (0.83) |
|  | Median (quartiles) | 1.56 (1.14,2.1) |
|  | Range | 0.15-14.5 |
| SHBG [nmol/l] | Mean (SD) | 53.92 (31.33) |
|  | Median (quartiles) | 48.3 (32.4,68.02) |
|  | Range | 4.6-239 |
| FAI [n] | Mean (SD) | 4.57 (6.07) |
|  | Median (quartiles) | 3.17 (1.98,5.51) |
|  | Range | 0.17-139.42 |
| DHEA-S [μmol/l] | Mean (SD) | 8.53 (3.68) |
|  | Median (quartiles) | 8 (5.98,10.3) |
|  | Range | 1.1-27.1 |
| TSH [μIU/ml] | Mean (SD) | 2.19 (2.6) |
|  | Median (quartiles) | 1.9 (1.38,2.6) |
|  | Range | 0-63.2 |
| fT3 [pmol/l] | Mean (SD) | 5.44 (1.54) |
|  | Median (quartiles) | 5.42 (4.86,5.92) |
|  | Range | 2.25-38.3 |
| fT4 [pmol/l] | Mean (SD) | 16.33 (3.72) |
|  | Median (quartiles) | 16 (14.6,17.7) |
|  | Range | 2.9-100 |
| anti-TPO [IU/ml] | Mean (SD) | 31.05 (75.93) |
|  | Median (quartiles) | 11.8 (9,16) |
|  | Range | 9-660 |
| anti-TG [IU/ml] | Mean (SD) | 56.68 (209.55) |
|  | Median (quartiles) | 16.2 (14.8,18.78) |
|  | Range | 11-4400 |
| Fasting glucose [mmol/l] | Mean (SD) | 4.79 (0.59) |
|  | Median (quartiles) | 4.71 (4.48,5) |
|  | Range | 3.24-12.1 |
| 120' 75OGTT glucose [mmol/l] | Mean (SD) | 5.35 (1.37) |
|  | Median (quartiles) | 5.22 (4.4,6.21) |
|  | Range | 2.11-13.4 |
| Fasting insulin [μU/ml] | Mean (SD) | 10.98 (8.88) |
|  | Median (quartiles) | 8.47 (5.84,12.7) |
|  | Range | 0.4-71.9 |
| 120' 75OGTT insulin [μU/ml] | Mean (SD) | 68.18 (65.65) |
|  | Median (quartiles) | 48.7 (30.78,82.7) |
|  | Range | 3.59-729 |
| HOMA-IR [n] | Mean (SD) | 2.42 (2.26) |
|  | Median (quartiles) | 1.78 (1.19,2.75) |
|  | Range | 0.12-24.16 |
| Total cholesterol [mmol/l] | Mean (SD) | 4.63 (0.85) |
|  | Median (quartiles) | 4.5 (4.1,5.1) |
|  | Range | 2.6-11 |
| HDL cholesterol [mmol/l] | Mean (SD) | 1.71 (0.42) |
|  | Median (quartiles) | 1.68 (1.42,1.95) |
|  | Range | 0.76-3.73 |
| LDL cholesterol [mmol/l] | Mean (SD) | 2.49 (0.73) |
|  | Median (quartiles) | 2.4 (2,2.9) |
|  | Range | 0.7-8 |
| Triglycerides [mmol/l] | Mean (SD) | 0.94 (0.5) |
|  | Median (quartiles) | 0.8 (0.62,1.11) |
|  | Range | 0.27,4.29 |
| CRP [mg/l] | Mean (SD) | 2.33 (3.56) |
|  | Median (quartiles) | 1 (0.6,2.28) |
|  | Range | 0,42.2 |

SD - standard deviation, quartiles: Q1(lower quartile), Q3 (upper quartile), Range: minimum value - maximum value, FSH - follicle-stimulating hormone, LH - luteinizing hormone, SHBG - sex hormone binding globulin, FAI - free androgen index, DHEA-S - dehydroepiandrosterone sulphate, TSH - thyroid-stimulating hormone, fT3 - free triiodothyronine, fT4 - free thyroxine, TPOAb - thyroid peroxidase antibodies, TGAb - thyroglobulin antibodies, OGTT - 75-g oral glucose tolerance test, HOMA-IR - homeostasis model assessment for insulin resistance, HDL - high-density lipoprotein, LDL - low-density lipoprotein, CRP - C-reactive protein

Supplementary Table 2. Impact of selected variables on Health-Related Quality of Life (HRQoL) in Physical Functioning domain among women with irregular menstruation.

| **Variable** | | **Univariate models** | | | | **Multivariate model** | | | |
| --- | --- | --- | --- | --- | --- | --- | --- | --- | --- |
|  |  | **Parameter** | **95%CI** | | **p** | **Parameter** | **95%CI** | | **p** |
| Age [years] | | -0.437 | -0.58 | -0.294 | <0.001 * | -0.203 | -0.351 | -0.056 | 0.007 * |
| mFG [n] | | -0.488 | -0.656 | -0.319 | <0.001 * | -0.248 | -0.415 | -0.082 | 0.004 * |
| LS | 0, I-1 | ref. |  |  |  | ref. |  |  |  |
|  | I-2 | -2.959 | -4.764 | -1.155 | 0.001 * | -2.432 | -4.107 | -0.757 | 0.004 * |
|  | I-3 | -7.526 | -10.515 | -4.536 | <0.001 * | -4.607 | -7.413 | -1.802 | 0.001 * |
|  | I-4, II-1, II-2 | -14.26 | -20.346 | -8.174 | <0.001 * | -12.177 | -17.821 | -6.532 | <0.001 * |
| BMI [kg/m²] | | -0.723 | -0.844 | -0.601 | <0.001 * | -0.282 | -0.472 | -0.092 | 0.004 * |
| Cycle length [days] | | 0.009 | -0.001 | 0.019 | 0.085 | -0.001 | -0.011 | 0.009 | 0.868 |
| Infertility | No | ref. |  |  |  |  |  |  |  |
|  | Yes | -1.565 | -4.134 | 1.004 | 0.232 |  |  |  |  |
| Dysmenorrhea | No | ref. |  |  |  | ref. |  |  |  |
|  | Yes | -3.364 | -4.962 | -1.766 | <0.001 * | -2.923 | -4.584 | -1.263 | 0.001 * |
| Abnormal uterine  bleeding | No | ref. |  |  |  | ref. |  |  |  |
|  | HMB | -4.237 | -6.005 | -2.469 | <0.001 * | -1.426 | -3.23 | 0.377 | 0.121 |
|  | IMB | -0.589 | -3.617 | 2.439 | 0.703 | 1.163 | -1.632 | 3.959 | 0.414 |
| Right Ovarian Volume [ml] | | -0.023 | -0.136 | 0.09 | 0.688 |  |  |  |  |
| Left Ovarian Volume [ml] | | 0.039 | -0.037 | 0.116 | 0.317 |  |  |  |  |
| Polycystic ovarian morphology | No | ref. |  |  |  |  |  |  |  |
|  | Yes | 1.033 | -0.587 | 2.653 | 0.211 |  |  |  |  |
| Dominant follicle | No | ref. |  |  |  |  |  |  |  |
|  | Yes | 0.256 | -1.93 | 2.442 | 0.818 |  |  |  |  |
| Corpus Luteum | No | ref. |  |  |  |  |  |  |  |
|  | Yes | 1.143 | -1.563 | 3.849 | 0.407 |  |  |  |  |
| Simple Cyst | No | ref. |  |  |  |  |  |  |  |
|  | Yes | 1.012 | -3.125 | 5.148 | 0.631 |  |  |  |  |
| Ovarian lesion | No | ref. |  |  |  |  |  |  |  |
|  | Yes | -1.969 | -5.17 | 1.233 | 0.228 |  |  |  |  |
| Anti-Müllerian hormone [pmol/l] | | 0.034 | 0.01 | 0.058 | 0.005 * | 0.029 | 0.004 | 0.053 | 0.021 * |
| FSH [mIU/ml] | | -0.096 | -0.198 | 0.005 | 0.063 | -0.089 | -0.184 | 0.006 | 0.067 |
| LH [mIU/ml] | | -0.019 | -0.089 | 0.052 | 0.603 |  |  |  |  |
| Estradiol [pmol/l] | | 0 | -0.003 | 0.002 | 0.696 |  |  |  |  |
| Prolactin [μIU/ml] | | 0 | -0.002 | 0.003 | 0.806 |  |  |  |  |
| Testosterone [nmol/l] | | -0.811 | -1.784 | 0.162 | 0.102 | -1.041 | -2.424 | 0.343 | 0.14 |
| SHBG [nmol/l] | | 0.053 | 0.027 | 0.078 | <0.001 * | -0.022 | -0.052 | 0.008 | 0.151 |
| FAI [n] | | -0.246 | -0.378 | -0.115 | <0.001 * | 0.094 | -0.12 | 0.308 | 0.389 |
| DHEA-S [μmol/l] | | 0.079 | -0.14 | 0.297 | 0.481 |  |  |  |  |
| TSH [μIU/ml] | | 0.125 | -0.185 | 0.435 | 0.43 |  |  |  |  |
| fT3 [pmol/l] | | -0.41 | -0.933 | 0.114 | 0.125 | -0.085 | -0.922 | 0.752 | 0.841 |
| fT4 [pmol/l] | | 0.083 | -0.133 | 0.299 | 0.451 |  |  |  |  |
| anti-TPO [IU/ml] | | -0.001 | -0.012 | 0.009 | 0.818 |  |  |  |  |
| anti-TG [IU/ml] | | -0.001 | -0.005 | 0.002 | 0.484 |  |  |  |  |
| Fasting glucose [mmol/l] | | -3.208 | -4.569 | -1.847 | <0.001 * | 0.494 | -1.918 | 2.906 | 0.688 |
| 120' 75OGTT glucose [mmol/l] | | -1.222 | -1.806 | -0.638 | <0.001 * | 0.338 | -0.357 | 1.032 | 0.34 |
| Fasting insulin [μU/ml] | | -0.421 | -0.508 | -0.334 | <0.001 * | 0.058 | -0.538 | 0.653 | 0.85 |
| 120' 75OGTT insulin [μU/ml] | | -0.036 | -0.048 | -0.024 | <0.001 * | -0.004 | -0.022 | 0.015 | 0.683 |
| HOMA-IR [n] | | -1.669 | -2.011 | -1.327 | <0.001 * | -0.671 | -3.219 | 1.878 | 0.606 |
| Total cholesterol [mmol/l] | | -0.564 | -1.51 | 0.382 | 0.242 |  |  |  |  |
| HDL cholesterol [mmol/l] | | 6.966 | 5.079 | 8.854 | <0.001 * | 1.815 | -0.341 | 3.971 | 0.099 |
| LDL cholesterol [mmol/l] | | -1.625 | -2.719 | -0.532 | 0.004 * | -0.238 | -1.331 | 0.855 | 0.669 |
| Triglycerides [mmol/l] | | -6.781 | -8.342 | -5.221 | <0.001 * | -2.268 | -4.08 | -0.457 | 0.014 * |
| CRP [mg/l] | | -0.973 | -1.191 | -0.755 | <0.001 * | -0.454 | -0.692 | -0.217 | <0.001 * |

95%CI - 95% confidence interval, * statistically significant (p<0.05), mFG - modified Ferriman-Gallwey scale score, LS - Ludwig scale score, BMI - body mass index, FSH - follicle-stimulating hormone, LH - luteinizing hormone, SHBG - sex hormone binding globulin, FAI - free androgen index, DHEA-S - dehydroepiandrosterone sulphate, TSH - thyroid-stimulating hormone, fT3 - free triiodothyronine, fT4 - free thyroxine, TPOAb - thyroid peroxidase antibodies, TGAb - thyroglobulin antibodies, 75OGTT - 75-g oral glucose tolerance test, HOMA-IR - homeostasis model assessment for insulin resistance, HDL - high-density lipoprotein, LDL - low-density lipoprotein, CRP - C-reactive protein

Supplementary Table 3. Impact of selected variables on Health-Related Quality of Life (HRQoL) in Role Limitations due to Physical Health domain among women with irregular menstruation.

| **Variable** | | **Univariate models** | | | | **Multivariate model** | | | |
| --- | --- | --- | --- | --- | --- | --- | --- | --- | --- |
|  |  | **Parameter** | **95%CI** | | **p** | **Parameter** | **95%CI** | | **p** |
| Age [years] | | -0.623 | -0.992 | -0.254 | 0.001 * | -0.427 | -0.819 | -0.036 | 0.032 * |
| mFG | | -0.791 | -1.225 | -0.356 | <0.001 * | -0.573 | -1.024 | -0.123 | 0.013 * |
| LS | 0, I-1 | ref. |  |  |  | ref. |  |  |  |
|  | I-2 | -6.059 | -10.711 | -1.406 | 0.011 * | -4.708 | -9.332 | -0.084 | 0.046 * |
|  | I-3 | -12.419 | -20.127 | -4.71 | 0.002 * | -7.948 | -15.664 | -0.232 | 0.044 * |
|  | I-4, II-1, II-2 | -31.46 | -47.151 | -15.768 | <0.001 * | -26.625 | -42.152 | -11.099 | 0.001 * |
| BMI [kg/m²] | | -0.543 | -0.872 | -0.214 | 0.001 * | 0.07 | -0.428 | 0.567 | 0.783 |
| Cycle length [days] | | 0.021 | -0.004 | 0.046 | 0.099 | -0.013 | -0.04 | 0.013 | 0.323 |
| Infertility | No | ref. |  |  |  |  |  |  |  |
|  | Yes | 0.68 | -5.884 | 7.244 | 0.839 |  |  |  |  |
| Dysmenorrhea | No | ref. |  |  |  | ref. |  |  |  |
|  | Yes | -11.962 | -16.009 | -7.915 | <0.001 * | -10.933 | -15.49 | -6.377 | <0.001 * |
| Abnormal uterine  bleeding | No | ref. |  |  |  | ref. |  |  |  |
|  | HMB | -9.803 | -14.326 | -5.28 | <0.001 * | -3.761 | -8.722 | 1.199 | 0.137 |
|  | IMB | -5.502 | -13.249 | 2.244 | 0.164 | -0.061 | -7.756 | 7.634 | 0.988 |
| Right Ovarian Volume [ml] | | -0.052 | -0.341 | 0.237 | 0.724 |  |  |  |  |
| Left Ovarian Volume [ml] | | 0.05 | -0.146 | 0.245 | 0.618 |  |  |  |  |
| Polycystic ovarian morphology | No | ref. |  |  |  |  |  |  |  |
|  | Yes | 0.784 | -3.355 | 4.922 | 0.71 |  |  |  |  |
| Dominant follicle | No | ref. |  |  |  | ref. |  |  |  |
|  | Yes | -5.611 | -11.182 | -0.041 | 0.048 * | -3.484 | -9.174 | 2.207 | 0.23 |
| Corpus Luteum | No | ref. |  |  |  |  |  |  |  |
|  | Yes | 4.199 | -2.707 | 11.105 | 0.233 |  |  |  |  |
| Simple Cyst | No | ref. |  |  |  |  |  |  |  |
|  | Yes | 0.608 | -9.954 | 11.169 | 0.91 |  |  |  |  |
| Ovarian lesion | No | ref. |  |  |  |  |  |  |  |
|  | Yes | -1.264 | -9.443 | 6.915 | 0.762 |  |  |  |  |
| Anti-Müllerian hormone [pmol/l] | | 0.047 | -0.014 | 0.108 | 0.129 | 0.033 | -0.028 | 0.095 | 0.291 |
| FSH [mIU/ml] | | -0.051 | -0.31 | 0.208 | 0.698 |  |  |  |  |
| LH [mIU/ml] | | 0.072 | -0.109 | 0.253 | 0.434 |  |  |  |  |
| Estradiol [pmol/l] | | -0.005 | -0.011 | 0 | 0.073 | -0.004 | -0.009 | 0.002 | 0.221 |
| Prolactin [μIU/ml] | | -0.002 | -0.009 | 0.005 | 0.577 |  |  |  |  |
| Testosterone [nmol/l] | | 0.471 | -2.016 | 2.958 | 0.71 |  |  |  |  |
| SHBG [nmol/l] | | 0.025 | -0.041 | 0.09 | 0.463 |  |  |  |  |
| FAI [n] | | -0.055 | -0.393 | 0.283 | 0.75 |  |  |  |  |
| DHEA-S [μmol/l] | | 0.283 | -0.275 | 0.841 | 0.321 |  |  |  |  |
| TSH [μIU/ml] | | 0.022 | -0.769 | 0.813 | 0.956 |  |  |  |  |
| fT3 [pmol/l] | | 0.217 | -1.12 | 1.555 | 0.75 |  |  |  |  |
| fT4 [pmol/l] | | 0.107 | -0.445 | 0.658 | 0.705 |  |  |  |  |
| anti-TPO [IU/ml] | | -0.001 | -0.028 | 0.027 | 0.967 |  |  |  |  |
| anti-TG [IU/ml] | | 0.004 | -0.005 | 0.014 | 0.385 |  |  |  |  |
| Fasting glucose [mmol/l] | | -1.343 | -4.859 | 2.172 | 0.454 |  |  |  |  |
| 120' 75OGTT glucose [mmol/l] | | -0.85 | -2.361 | 0.661 | 0.27 |  |  |  |  |
| Fasting insulin [μU/ml] | | -0.311 | -0.542 | -0.08 | 0.008 * | -0.5 | -1.68 | 0.68 | 0.406 |
| 120' 75OGTT insulin [μU/ml] | | -0.037 | -0.068 | -0.005 | 0.021 * | -0.016 | -0.06 | 0.028 | 0.472 |
| HOMA-IR [n] | | -1.089 | -2 | -0.179 | 0.019 * | 2.096 | -2.515 | 6.708 | 0.373 |
| Total cholesterol [mmol/l] | | 0.318 | -2.098 | 2.735 | 0.796 |  |  |  |  |
| HDL cholesterol [mmol/l] | | 6.49 | 1.56 | 11.419 | 0.01 * | 1.037 | -4.782 | 6.856 | 0.727 |
| LDL cholesterol [mmol/l] | | -0.284 | -3.088 | 2.52 | 0.842 |  |  |  |  |
| Triglycerides [mmol/l] | | -6.365 | -10.474 | -2.256 | 0.002 * | -2.657 | -7.508 | 2.195 | 0.283 |
| CRP [mg/l] | | -0.858 | -1.434 | -0.283 | 0.004 * | -0.685 | -1.337 | -0.032 | 0.04 * |

95%CI - 95% confidence interval, * statistically significant (p<0.05), mFG - modified Ferriman-Gallwey scale score, LS - Ludwig scale score, BMI - body mass index, FSH - follicle-stimulating hormone, LH - luteinizing hormone, SHBG - sex hormone binding globulin, FAI - free androgen index, DHEA-S - dehydroepiandrosterone sulphate, TSH - thyroid-stimulating hormone, fT3 - free triiodothyronine, fT4 - free thyroxine, TPOAb - thyroid peroxidase antibodies, TGAb - thyroglobulin antibodies, 75OGTT - 75-g oral glucose tolerance test, HOMA-IR - homeostasis model assessment for insulin resistance, HDL - high-density lipoprotein, LDL - low-density lipoprotein, CRP - C-reactive protein

Supplementary Table 4. Impact of selected variables on Health-Related Quality of Life (HRQoL) in Role Limitations due to Emotional Problems domain among women with irregular menstruation.

| **Variable** | | **Univariate models** | | | | **Multivariate model** | | | |
| --- | --- | --- | --- | --- | --- | --- | --- | --- | --- |
|  |  | **Parameter** | **95%CI** | | **p** | **Parameter** | **95%CI** | | **p** |
| Age [years] | | -0.113 | -0.553 | 0.326 | 0.612 |  |  |  |  |
| mFG [n] | | -0.788 | -1.303 | -0.272 | 0.003 * | -0.588 | -1.125 | -0.051 | 0.032 * |
| LS | 0, I-1 | ref. |  |  |  | ref. |  |  |  |
|  | I-2 | -3.078 | -8.644 | 2.489 | 0.278 | -1.321 | -6.921 | 4.278 | 0.643 |
|  | I-3 | -6.159 | -15.382 | 3.064 | 0.19 | -3.699 | -12.997 | 5.599 | 0.435 |
|  | I-4, II-1, II-2 | -17.101 | -35.875 | 1.673 | 0.074 | -12.798 | -31.554 | 5.958 | 0.181 |
| BMI [kg/m²] | | -0.554 | -0.944 | -0.164 | 0.005 * | -0.225 | -0.726 | 0.276 | 0.379 |
| Cycle length [days] | | 0.02 | -0.01 | 0.049 | 0.198 | 0.005 | -0.027 | 0.036 | 0.779 |
| Infertility | No | ref. |  |  |  | ref. |  |  |  |
|  | Yes | 6.679 | -1.078 | 14.435 | 0.091 | 8.448 | 0.652 | 16.245 | 0.034 * |
| Dysmenorrhea | No | ref. |  |  |  | ref. |  |  |  |
|  | Yes | -6.798 | -11.65 | -1.947 | 0.006 * | -5.369 | -10.86 | 0.122 | 0.055 |
| Abnormal uterine  bleeding | No | ref. |  |  |  | ref. |  |  |  |
|  | HMB | -6.927 | -12.309 | -1.545 | 0.012 * | -3.123 | -9.125 | 2.88 | 0.308 |
|  | IMB | -7.198 | -16.415 | 2.02 | 0.126 | -4.501 | -13.803 | 4.801 | 0.343 |
| Right Ovarian Volume [ml] | | 0.061 | -0.282 | 0.403 | 0.728 |  |  |  |  |
| Left Ovarian Volume [ml] | | 0.034 | -0.197 | 0.266 | 0.771 |  |  |  |  |
| Polycystic ovarian morphology | No | ref. |  |  |  |  |  |  |  |
|  | Yes | -1.192 | -6.09 | 3.705 | 0.633 |  |  |  |  |
| Dominant follicle | No | ref. |  |  |  |  |  |  |  |
|  | Yes | 0.428 | -6.177 | 7.033 | 0.899 |  |  |  |  |
| Corpus Luteum | No | ref. |  |  |  |  |  |  |  |
|  | Yes | 4.556 | -3.618 | 12.729 | 0.274 |  |  |  |  |
| Simple Cyst | No | ref. |  |  |  |  |  |  |  |
|  | Yes | 5.167 | -7.327 | 17.662 | 0.417 |  |  |  |  |
| Ovarian lesion | No | ref. |  |  |  |  |  |  |  |
|  | Yes | 1.739 | -7.94 | 11.417 | 0.725 |  |  |  |  |
| Anti-Müllerian hormone [pmol/l] | | -0.018 | -0.09 | 0.054 | 0.62 |  |  |  |  |
| FSH [mIU/ml] | | 0.205 | -0.101 | 0.512 | 0.19 | 0.063 | -0.291 | 0.416 | 0.728 |
| LH [mIU/ml] | | 0.159 | -0.054 | 0.373 | 0.143 | 0.117 | -0.126 | 0.359 | 0.345 |
| Estradiol [pmol/l] | | 0.003 | -0.003 | 0.01 | 0.314 |  |  |  |  |
| Prolactin [μIU/ml] | | 0.003 | -0.006 | 0.011 | 0.535 |  |  |  |  |
| Testosterone [nmol/l] | | -0.38 | -3.324 | 2.564 | 0.8 |  |  |  |  |
| SHBG [nmol/l] | | 0.042 | -0.036 | 0.12 | 0.288 |  |  |  |  |
| FAI [n] | | -0.109 | -0.51 | 0.291 | 0.592 |  |  |  |  |
| DHEA-S [μmol/l] | | 0.371 | -0.289 | 1.032 | 0.27 |  |  |  |  |
| TSH [μIU/ml] | | 0.34 | -0.596 | 1.276 | 0.476 |  |  |  |  |
| fT3 [pmol/l] | | 0.884 | -0.698 | 2.466 | 0.273 |  |  |  |  |
| fT4 [pmol/l] | | 0.195 | -0.458 | 0.848 | 0.557 |  |  |  |  |
| anti-TPO [IU/ml] | | -0.015 | -0.047 | 0.017 | 0.357 |  |  |  |  |
| anti-TG [IU/ml] | | 0.005 | -0.007 | 0.017 | 0.401 |  |  |  |  |
| Fasting glucose [mmol/l] | | 1.222 | -2.936 | 5.379 | 0.564 |  |  |  |  |
| 120' 75OGTT glucose [mmol/l] | | 0.735 | -1.053 | 2.523 | 0.42 |  |  |  |  |
| Fasting insulin [μU/ml] | | -0.061 | -0.335 | 0.213 | 0.663 |  |  |  |  |
| 120' 75OGTT insulin [μU/ml] | | -0.017 | -0.054 | 0.02 | 0.365 |  |  |  |  |
| HOMA-IR [n] | | -0.232 | -1.311 | 0.848 | 0.674 |  |  |  |  |
| Total cholesterol [mmol/l] | | 0.536 | -2.324 | 3.396 | 0.713 |  |  |  |  |
| HDL cholesterol [mmol/l] | | 2.326 | -3.525 | 8.177 | 0.436 |  |  |  |  |
| LDL cholesterol [mmol/l] | | 0.556 | -2.762 | 3.874 | 0.742 |  |  |  |  |
| Triglycerides [mmol/l] | | -3.222 | -8.103 | 1.659 | 0.196 | 0.311 | -5.199 | 5.821 | 0.912 |
| CRP [mg/l] | | -1.027 | -1.708 | -0.346 | 0.003 * | -0.841 | -1.622 | -0.06 | 0.035 * |

95%CI - 95% confidence interval, * statistically significant (p<0.05), mFG - modified Ferriman-Gallwey scale score, LS - Ludwig scale score, BMI - body mass index, FSH - follicle-stimulating hormone, LH - luteinizing hormone, SHBG - sex hormone binding globulin, FAI - free androgen index, DHEA-S - dehydroepiandrosterone sulphate, TSH - thyroid-stimulating hormone, fT3 - free triiodothyronine, fT4 - free thyroxine, TPOAb - thyroid peroxidase antibodies, TGAb - thyroglobulin antibodies, 75OGTT - 75-g oral glucose tolerance test, HOMA-IR - homeostasis model assessment for insulin resistance, HDL - high-density lipoprotein, LDL - low-density lipoprotein, CRP - C-reactive protein

Supplementary Table 5. Impact of selected variables on Health-Related Quality of Life (HRQoL) in Energy/Fatigue domain among women with irregular menstruation.

| **Variable** | | **Univariate models** | | | | **Multivariate model** | | | |
| --- | --- | --- | --- | --- | --- | --- | --- | --- | --- |
|  |  | **Parameter** | **95%CI** | | **p** | **Parameter** | **95%CI** | | **p** |
| Age [years] | | -0.115 | -0.307 | 0.078 | 0.242 |  |  |  |  |
| mFG | | -0.463 | -0.688 | -0.238 | <0.001 * | -0.375 | -0.611 | -0.139 | 0.002 * |
| LS | 0, I-1 | ref. |  |  |  | ref. |  |  |  |
|  | I-2 | -3.724 | -6.151 | -1.297 | 0.003 * | -2.891 | -5.311 | -0.47 | 0.019 * |
|  | I-3 | -6.163 | -10.184 | -2.142 | 0.003 * | -4.959 | -8.969 | -0.948 | 0.015 * |
|  | I-4, II-1, II-2 | -5.854 | -14.039 | 2.331 | 0.161 | -4.114 | -12.224 | 3.995 | 0.32 |
| BMI [kg/m²] | | -0.272 | -0.443 | -0.101 | 0.002 * | -0.121 | -0.385 | 0.142 | 0.367 |
| Cycle length [days] | | 0.016 | 0.003 | 0.029 | 0.019 * | 0.006 | -0.008 | 0.019 | 0.414 |
| Infertility | No | ref. |  |  |  |  |  |  |  |
|  | Yes | -0.96 | -4.365 | 2.445 | 0.58 |  |  |  |  |
| Dysmenorrhea | No | ref. |  |  |  | ref. |  |  |  |
|  | Yes | -4.735 | -6.85 | -2.62 | <0.001 * | -2.982 | -5.368 | -0.597 | 0.014 * |
| Abnormal uterine  bleeding | No | ref. |  |  |  | ref. |  |  |  |
|  | HMB | -5.861 | -8.201 | -3.521 | <0.001 * | -3.695 | -6.296 | -1.095 | 0.005 * |
|  | IMB | -1.378 | -5.386 | 2.63 | 0.5 | -0.204 | -4.217 | 3.809 | 0.921 |
| Right Ovarian Volume [ml] | | 0.027 | -0.123 | 0.178 | 0.72 |  |  |  |  |
| Left Ovarian Volume [ml] | | 0.07 | -0.031 | 0.171 | 0.175 | 0.077 | -0.022 | 0.177 | 0.127 |
| Polycystic ovarian morphology | No | ref. |  |  |  |  |  |  |  |
|  | Yes | -1.056 | -3.202 | 1.09 | 0.335 |  |  |  |  |
| Dominant follicle | No | ref. |  |  |  | ref. |  |  |  |
|  | Yes | 2.419 | -0.473 | 5.311 | 0.101 | 2.65 | -0.202 | 5.501 | 0.069 |
| Corpus Luteum | No | ref. |  |  |  |  |  |  |  |
|  | Yes | -0.516 | -4.102 | 3.069 | 0.778 |  |  |  |  |
| Simple Cyst | No | ref. |  |  |  |  |  |  |  |
|  | Yes | 2.283 | -3.195 | 7.762 | 0.414 |  |  |  |  |
| Ovarian lesion | No | ref. |  |  |  |  |  |  |  |
|  | Yes | -0.287 | -4.531 | 3.957 | 0.895 |  |  |  |  |
| Anti-Müllerian hormone [pmol/l] | | 0.006 | -0.026 | 0.038 | 0.709 |  |  |  |  |
| FSH [mIU/ml] | | -0.002 | -0.137 | 0.132 | 0.974 |  |  |  |  |
| LH [mIU/ml] | | 0.026 | -0.068 | 0.119 | 0.592 |  |  |  |  |
| Estradiol [pmol/l] | | 0.001 | -0.002 | 0.004 | 0.507 |  |  |  |  |
| Prolactin [μIU/ml] | | 0 | -0.004 | 0.003 | 0.932 |  |  |  |  |
| Testosterone [nmol/l] | | -0.42 | -1.71 | 0.87 | 0.523 |  |  |  |  |
| SHBG [nmol/l] | | 0.028 | -0.006 | 0.062 | 0.103 | -0.014 | -0.053 | 0.025 | 0.489 |
| FAI [n] | | -0.081 | -0.257 | 0.094 | 0.363 |  |  |  |  |
| DHEA-S [μmol/l] | | -0.016 | -0.306 | 0.274 | 0.914 |  |  |  |  |
| TSH [μIU/ml] | | 0.083 | -0.327 | 0.494 | 0.691 |  |  |  |  |
| fT3 [pmol/l] | | -0.517 | -1.211 | 0.176 | 0.144 | -0.31 | -1.013 | 0.393 | 0.387 |
| fT4 [pmol/l] | | -0.108 | -0.395 | 0.178 | 0.458 |  |  |  |  |
| anti-TPO [IU/ml] | | -0.006 | -0.02 | 0.008 | 0.438 |  |  |  |  |
| anti-TG [IU/ml] | | 0.001 | -0.004 | 0.006 | 0.779 |  |  |  |  |
| Fasting glucose [mmol/l] | | -0.206 | -2.031 | 1.619 | 0.825 |  |  |  |  |
| 120' 75OGTT glucose [mmol/l] | | -0.258 | -1.04 | 0.524 | 0.517 |  |  |  |  |
| Fasting insulin [μU/ml] | | -0.11 | -0.23 | 0.01 | 0.072 | -0.052 | -0.61 | 0.506 | 0.856 |
| 120' 75OGTT insulin [μU/ml] | | -0.01 | -0.027 | 0.006 | 0.21 |  |  |  |  |
| HOMA-IR [n] | | -0.395 | -0.868 | 0.079 | 0.102 | 0.464 | -1.696 | 2.624 | 0.673 |
| Total cholesterol [mmol/l] | | 0.042 | -1.212 | 1.296 | 0.947 |  |  |  |  |
| HDL cholesterol [mmol/l] | | 4.306 | 1.754 | 6.859 | 0.001 * | 2.963 | -0.101 | 6.026 | 0.058 |
| LDL cholesterol [mmol/l] | | -0.959 | -2.413 | 0.494 | 0.196 | -1.391 | -2.924 | 0.142 | 0.075 |
| Triglycerides [mmol/l] | | -1.694 | -3.833 | 0.445 | 0.121 | 0.977 | -1.603 | 3.558 | 0.458 |
| CRP [mg/l] | | -0.321 | -0.62 | -0.022 | 0.036 * | -0.102 | -0.441 | 0.237 | 0.554 |

95%CI - 95% confidence interval, * statistically significant (p<0.05), mFG - modified Ferriman-Gallwey scale score, LS - Ludwig scale score, BMI - body mass index, FSH - follicle-stimulating hormone, LH - luteinizing hormone, SHBG - sex hormone binding globulin, FAI - free androgen index, DHEA-S - dehydroepiandrosterone sulphate, TSH - thyroid-stimulating hormone, fT3 - free triiodothyronine, fT4 - free thyroxine, TPOAb - thyroid peroxidase antibodies, TGAb - thyroglobulin antibodies, 75OGTT - 75-g oral glucose tolerance test, HOMA-IR - homeostasis model assessment for insulin resistance, HDL - high-density lipoprotein, LDL - low-density lipoprotein, CRP - C-reactive protein

Supplementary Table 6. Impact of selected variables on Health-Related Quality of Life (HRQoL) in Emotional Well-Being domain among women with irregular menstruation.

| **Variable** | | **Univariate models** | | | | **Multivariate model** | | | |
| --- | --- | --- | --- | --- | --- | --- | --- | --- | --- |
|  |  | **Parameter** | **95%CI** | | **p** | **Parameter** | **95%CI** | | **p** |
| Age [years] | | -0.172 | -0.354 | 0.01 | 0.064 | -0.072 | -0.264 | 0.12 | 0.462 |
| mFG | | -0.402 | -0.616 | -0.189 | <0.001 * | -0.358 | -0.582 | -0.134 | 0.002 * |
| LS | 0, I-1 | ref. |  |  |  | ref. |  |  |  |
|  | I-2 | -1.524 | -3.829 | 0.78 | 0.195 | -0.586 | -2.889 | 1.718 | 0.618 |
|  | I-3 | -5.666 | -9.484 | -1.848 | 0.004 * | -3.845 | -7.684 | -0.006 | 0.05 * |
|  | I-4, II-1, II-2 | -5.024 | -12.796 | 2.747 | 0.205 | -2.834 | -10.586 | 4.917 | 0.473 |
| BMI [kg/m²] | | -0.298 | -0.459 | -0.136 | <0.001 * | -0.247 | -0.5 | 0.005 | 0.055 |
| Cycle length [days] | | 0.006 | -0.006 | 0.019 | 0.308 |  |  |  |  |
| Infertility | No | ref. |  |  |  |  |  |  |  |
|  | Yes | 0.62 | -2.603 | 3.844 | 0.706 |  |  |  |  |
| Dysmenorrhea | No | ref. |  |  |  | ref. |  |  |  |
|  | Yes | -2.805 | -4.819 | -0.792 | 0.006 * | -2.24 | -4.455 | -0.025 | 0.047 * |
| Abnormal uterine  bleeding | No | ref. |  |  |  | ref. |  |  |  |
|  | HMB | -3.407 | -5.638 | -1.176 | 0.003 * | -2.061 | -4.521 | 0.398 | 0.1 |
|  | IMB | -2.743 | -6.564 | 1.078 | 0.159 | -1.716 | -5.538 | 2.106 | 0.379 |
| Right Ovarian Volume [ml] | | 0.056 | -0.086 | 0.199 | 0.435 |  |  |  |  |
| Left Ovarian Volume [ml] | | 0.083 | -0.013 | 0.179 | 0.091 | 0.087 | -0.008 | 0.181 | 0.072 |
| Polycystic ovarian morphology | No | ref. |  |  |  |  |  |  |  |
|  | Yes | 0.618 | -1.414 | 2.65 | 0.551 |  |  |  |  |
| Dominant follicle | No | ref. |  |  |  |  |  |  |  |
|  | Yes | -0.067 | -2.809 | 2.674 | 0.961 |  |  |  |  |
| Corpus Luteum | No | ref. |  |  |  |  |  |  |  |
|  | Yes | 1.441 | -1.952 | 4.834 | 0.405 |  |  |  |  |
| Simple Cyst | No | ref. |  |  |  |  |  |  |  |
|  | Yes | 1.992 | -3.193 | 7.178 | 0.451 |  |  |  |  |
| Ovarian lesion | No | ref. |  |  |  |  |  |  |  |
|  | Yes | -0.94 | -4.957 | 3.076 | 0.646 |  |  |  |  |
| Anti-Müllerian hormone [pmol/l] | | 0.01 | -0.02 | 0.039 | 0.533 |  |  |  |  |
| FSH [mIU/ml] | | 0.017 | -0.11 | 0.144 | 0.792 |  |  |  |  |
| LH [mIU/ml] | | 0.024 | -0.064 | 0.113 | 0.591 |  |  |  |  |
| Estradiol [pmol/l] | | 0 | -0.002 | 0.003 | 0.866 |  |  |  |  |
| Prolactin [μIU/ml] | | 0.002 | -0.001 | 0.005 | 0.228 |  |  |  |  |
| Testosterone [nmol/l] | | 0.037 | -1.184 | 1.259 | 0.952 |  |  |  |  |
| SHBG [nmol/l] | | 0.025 | -0.008 | 0.057 | 0.136 | -0.021 | -0.058 | 0.017 | 0.278 |
| FAI [n] | | -0.028 | -0.194 | 0.138 | 0.743 |  |  |  |  |
| DHEA-S [μmol/l] | | 0.075 | -0.2 | 0.349 | 0.593 |  |  |  |  |
| TSH [μIU/ml] | | 0.146 | -0.243 | 0.534 | 0.462 |  |  |  |  |
| fT3 [pmol/l] | | 0.515 | -0.141 | 1.171 | 0.124 | 0.437 | -0.302 | 1.177 | 0.246 |
| fT4 [pmol/l] | | 0.162 | -0.109 | 0.433 | 0.241 |  |  |  |  |
| anti-TPO [IU/ml] | | -0.006 | -0.019 | 0.007 | 0.368 |  |  |  |  |
| anti-TG [IU/ml] | | -0.001 | -0.006 | 0.004 | 0.686 |  |  |  |  |
| Fasting glucose [mmol/l] | | 1.266 | -0.46 | 2.991 | 0.15 | 2.917 | 0.204 | 5.63 | 0.035 * |
| 120' 75OGTT glucose [mmol/l] | | 0.396 | -0.344 | 1.135 | 0.294 |  |  |  |  |
| Fasting insulin [μU/ml] | | -0.12 | -0.233 | -0.007 | 0.038 * | 0.135 | -0.542 | 0.811 | 0.696 |
| 120' 75OGTT insulin [μU/ml] | | 0.001 | -0.014 | 0.017 | 0.875 |  |  |  |  |
| HOMA-IR [n] | | -0.41 | -0.858 | 0.037 | 0.072 | -0.583 | -3.381 | 2.214 | 0.682 |
| Total cholesterol [mmol/l] | | 0.346 | -0.84 | 1.533 | 0.567 |  |  |  |  |
| HDL cholesterol [mmol/l] | | 2.087 | -0.338 | 4.513 | 0.092 | 0.173 | -2.657 | 3.004 | 0.904 |
| LDL cholesterol [mmol/l] | | 0.03 | -1.347 | 1.407 | 0.966 |  |  |  |  |
| Triglycerides [mmol/l] | | -1.246 | -3.272 | 0.78 | 0.228 |  |  |  |  |
| CRP [mg/l] | | -0.47 | -0.753 | -0.188 | 0.001 * | -0.301 | -0.623 | 0.022 | 0.068 |

95%CI - 95% confidence interval, * statistically significant (p<0.05), mFG - modified Ferriman-Gallwey scale score, LS - Ludwig scale score, BMI - body mass index, FSH - follicle-stimulating hormone, LH - luteinizing hormone, SHBG - sex hormone binding globulin, FAI - free androgen index, DHEA-S - dehydroepiandrosterone sulphate, TSH - thyroid-stimulating hormone, fT3 - free triiodothyronine, fT4 - free thyroxine, TPOAb - thyroid peroxidase antibodies, TGAb - thyroglobulin antibodies, 75OGTT - 75-g oral glucose tolerance test, HOMA-IR - homeostasis model assessment for insulin resistance, HDL - high-density lipoprotein, LDL - low-density lipoprotein, CRP - C-reactive protein

Supplementary Table 7. Impact of selected variables on Health-Related Quality of Life (HRQoL) in Social Functioning domain among women with irregular menstruation.

| **Variable** | | **Univariate models** | | | | **Multivariate model** | | | |
| --- | --- | --- | --- | --- | --- | --- | --- | --- | --- |
|  |  | **Parameter** | **95%CI** | | **p** | **Parameter** | **95%CI** | | **p** |
| Age [years] | | -0.074 | -0.353 | 0.205 | 0.602 |  |  |  |  |
| mFG [n] | | -0.72 | -1.046 | -0.395 | <0.001 * | -0.561 | -0.898 | -0.225 | 0.001 * |
| LS | 0, I-1 | ref. |  |  |  | ref. |  |  |  |
|  | I-2 | -4.963 | -8.463 | -1.463 | 0.005 * | -3.633 | -7.14 | -0.126 | 0.042 * |
|  | I-3 | -10.156 | -15.955 | -4.357 | 0.001 * | -7.972 | -13.78 | -2.163 | 0.007 * |
|  | I-4, II-1, II-2 | -17.958 | -29.761 | -6.155 | 0.003 * | -14.904 | -26.639 | -3.169 | 0.013 * |
| BMI [kg/m²] | | -0.309 | -0.557 | -0.061 | 0.015 * | -0.029 | -0.397 | 0.339 | 0.877 |
| Cycle length [days] | | 0.017 | -0.002 | 0.036 | 0.081 | 0.001 | -0.019 | 0.021 | 0.915 |
| Infertility | No | ref. |  |  |  |  |  |  |  |
|  | Yes | 1.748 | -3.179 | 6.676 | 0.486 |  |  |  |  |
| Dysmenorrhea | No | ref. |  |  |  | ref. |  |  |  |
|  | Yes | -5.556 | -8.626 | -2.485 | <0.001 * | -3.578 | -7.017 | -0.139 | 0.041 * |
| Abnormal uterine  bleeding | No | ref. |  |  |  | ref. |  |  |  |
|  | HMB | -6.685 | -10.082 | -3.288 | <0.001 * | -4.561 | -8.324 | -0.797 | 0.018 * |
|  | IMB | -7.373 | -13.191 | -1.555 | 0.013 * | -5.491 | -11.301 | 0.32 | 0.064 |
| Right Ovarian Volume [ml] | | -0.065 | -0.282 | 0.152 | 0.556 |  |  |  |  |
| Left Ovarian Volume [ml] | | 0.073 | -0.074 | 0.219 | 0.332 |  |  |  |  |
| Polycystic ovarian morphology | No | ref. |  |  |  |  |  |  |  |
|  | Yes | -0.577 | -3.685 | 2.53 | 0.715 |  |  |  |  |
| Dominant follicle | No | ref. |  |  |  |  |  |  |  |
|  | Yes | 2.076 | -2.113 | 6.266 | 0.331 |  |  |  |  |
| Corpus Luteum | No | ref. |  |  |  |  |  |  |  |
|  | Yes | 0.864 | -4.325 | 6.053 | 0.744 |  |  |  |  |
| Simple Cyst | No | ref. |  |  |  |  |  |  |  |
|  | Yes | 1.733 | -6.197 | 9.663 | 0.668 |  |  |  |  |
| Ovarian lesion | No | ref. |  |  |  |  |  |  |  |
|  | Yes | 2.35 | -3.79 | 8.49 | 0.453 |  |  |  |  |
| Anti-Müllerian hormone [pmol/l] | | 0.005 | -0.041 | 0.051 | 0.826 |  |  |  |  |
| FSH [mIU/ml] | | 0.088 | -0.106 | 0.283 | 0.374 |  |  |  |  |
| LH [mIU/ml] | | 0.118 | -0.018 | 0.253 | 0.088 | 0.087 | -0.046 | 0.22 | 0.198 |
| Estradiol [pmol/l] | | 0.002 | -0.002 | 0.006 | 0.34 |  |  |  |  |
| Prolactin [μIU/ml] | | 0.004 | -0.002 | 0.009 | 0.172 | 0.004 | -0.001 | 0.009 | 0.111 |
| Testosterone [nmol/l] | | -0.638 | -2.505 | 1.23 | 0.503 |  |  |  |  |
| SHBG [nmol/l] | | 0.016 | -0.034 | 0.065 | 0.535 |  |  |  |  |
| FAI [n] | | -0.06 | -0.314 | 0.194 | 0.643 |  |  |  |  |
| DHEA-S [μmol/l] | | -0.016 | -0.436 | 0.403 | 0.939 |  |  |  |  |
| TSH [μIU/ml] | | 0.167 | -0.427 | 0.761 | 0.581 |  |  |  |  |
| fT3 [pmol/l] | | 0.458 | -0.546 | 1.462 | 0.371 |  |  |  |  |
| fT4 [pmol/l] | | 0.205 | -0.209 | 0.619 | 0.332 |  |  |  |  |
| anti-TPO [IU/ml] | | -0.012 | -0.032 | 0.008 | 0.245 |  |  |  |  |
| anti-TG [IU/ml] | | 0 | -0.007 | 0.008 | 0.957 |  |  |  |  |
| Fasting glucose [mmol/l] | | -0.438 | -3.081 | 2.205 | 0.745 |  |  |  |  |
| 120' 75OGTT glucose [mmol/l] | | 0.54 | -0.591 | 1.671 | 0.349 |  |  |  |  |
| Fasting insulin [μU/ml] | | -0.128 | -0.301 | 0.046 | 0.15 | 0.557 | -0.247 | 1.361 | 0.174 |
| 120' 75OGTT insulin [μU/ml] | | -0.008 | -0.032 | 0.015 | 0.495 |  |  |  |  |
| HOMA-IR [n] | | -0.582 | -1.268 | 0.103 | 0.096 | -2.026 | -5.145 | 1.093 | 0.203 |
| Total cholesterol [mmol/l] | | 0.157 | -1.658 | 1.971 | 0.866 |  |  |  |  |
| HDL cholesterol [mmol/l] | | 2.432 | -1.279 | 6.142 | 0.199 | -0.073 | -4.427 | 4.282 | 0.974 |
| LDL cholesterol [mmol/l] | | -0.096 | -2.202 | 2.009 | 0.928 |  |  |  |  |
| Triglycerides [mmol/l] | | -2.281 | -5.378 | 0.815 | 0.149 | -0.178 | -3.816 | 3.461 | 0.924 |
| CRP [mg/l] | | -0.681 | -1.113 | -0.249 | 0.002 * | -0.53 | -1.019 | -0.04 | 0.034 * |

95%CI - 95% confidence interval, * statistically significant (p<0.05), mFG - modified Ferriman-Gallwey scale score, LS - Ludwig scale score, BMI - body mass index, FSH - follicle-stimulating hormone, LH - luteinizing hormone, SHBG - sex hormone binding globulin, FAI - free androgen index, DHEA-S - dehydroepiandrosterone sulphate, TSH - thyroid-stimulating hormone, fT3 - free triiodothyronine, fT4 - free thyroxine, TPOAb - thyroid peroxidase antibodies, TGAb - thyroglobulin antibodies, 75OGTT - 75-g oral glucose tolerance test, HOMA-IR - homeostasis model assessment for insulin resistance, HDL - high-density lipoprotein, LDL - low-density lipoprotein, CRP - C-reactive protein

Supplementary Table 8. Impact of selected variables on Health-Related Quality of Life (HRQoL) in Pain domain among women with irregular menstruation.

| **Variable** | | **Univariate models** | | | | **Multivariate model** | | | |
| --- | --- | --- | --- | --- | --- | --- | --- | --- | --- |
|  |  | **Parameter** | **95%CI** | | **p** | **Parameter** | **95%CI** | | **p** |
| Age [years] | | -0.076 | -0.337 | 0.186 | 0.57 |  |  |  |  |
| mFG [n] | | -0.359 | -0.666 | -0.052 | 0.022 * | -0.302 | -0.612 | 0.008 | 0.056 |
| LS | 0, I-1 | ref. |  |  |  | ref. |  |  |  |
|  | I-2 | -3.094 | -6.398 | 0.211 | 0.066 | -2.066 | -5.295 | 1.164 | 0.21 |
|  | I-3 | -6.054 | -11.529 | -0.58 | 0.03 * | -4.473 | -9.799 | 0.854 | 0.1 |
|  | I-4, II-1, II-2 | -9.059 | -20.202 | 2.085 | 0.111 | -7.15 | -17.962 | 3.663 | 0.195 |
| BMI [kg/m²] | | -0.11 | -0.343 | 0.123 | 0.353 |  |  |  |  |
| Cycle length [days] | | 0.021 | 0.003 | 0.039 | 0.02 * | -0.007 | -0.025 | 0.011 | 0.455 |
| Infertility | No | ref. |  |  |  |  |  |  |  |
|  | Yes | 0.217 | -4.402 | 4.836 | 0.927 |  |  |  |  |
| Dysmenorrhea | No | ref. |  |  |  | ref. |  |  |  |
|  | Yes | -11.829 | -14.629 | -9.029 | <0.001 * | -9.743 | -12.923 | -6.563 | <0.001 * |
| Abnormal uterine  bleeding | No | ref. |  |  |  | ref. |  |  |  |
|  | HMB | -9.739 | -12.893 | -6.585 | <0.001 * | -4.621 | -8.083 | -1.159 | 0.009 * |
|  | IMB | -3.893 | -9.295 | 1.509 | 0.158 | -1.356 | -6.722 | 4.01 | 0.62 |
| Right Ovarian Volume [ml] | | 0.014 | -0.189 | 0.218 | 0.892 |  |  |  |  |
| Left Ovarian Volume [ml] | | 0.115 | -0.022 | 0.253 | 0.1 | 0.124 | -0.013 | 0.261 | 0.076 |
| Polycystic ovarian morphology | No | ref. |  |  |  |  |  |  |  |
|  | Yes | 1.345 | -1.566 | 4.257 | 0.365 |  |  |  |  |
| Dominant follicle | No | ref. |  |  |  | ref. |  |  |  |
|  | Yes | -2.767 | -6.691 | 1.157 | 0.167 | -1.095 | -5.084 | 2.894 | 0.59 |
| Corpus Luteum | No | ref. |  |  |  |  |  |  |  |
|  | Yes | 1.833 | -3.029 | 6.695 | 0.46 |  |  |  |  |
| Simple Cyst | No | ref. |  |  |  |  |  |  |  |
|  | Yes | -1.077 | -8.509 | 6.355 | 0.776 |  |  |  |  |
| Ovarian lesion | No | ref. |  |  |  | ref. |  |  |  |
|  | Yes | -4.263 | -10.012 | 1.487 | 0.146 | -1.309 | -7.07 | 4.453 | 0.656 |
| Anti-Müllerian hormone [pmol/l] | | 0.045 | 0.002 | 0.088 | 0.039 * | 0.029 | -0.015 | 0.072 | 0.197 |
| FSH [mIU/ml] | | 0.002 | -0.181 | 0.184 | 0.984 |  |  |  |  |
| LH [mIU/ml] | | 0.018 | -0.109 | 0.145 | 0.779 |  |  |  |  |
| Estradiol [pmol/l] | | -0.003 | -0.007 | 0.001 | 0.169 | -0.002 | -0.006 | 0.002 | 0.394 |
| Prolactin [μIU/ml] | | 0.001 | -0.004 | 0.006 | 0.652 |  |  |  |  |
| Testosterone [nmol/l] | | 0.452 | -1.298 | 2.202 | 0.612 |  |  |  |  |
| SHBG [nmol/l] | | -0.007 | -0.053 | 0.039 | 0.763 |  |  |  |  |
| FAI [n] | | 0.016 | -0.222 | 0.254 | 0.893 |  |  |  |  |
| DHEA-S [μmol/l] | | 0.022 | -0.371 | 0.415 | 0.913 |  |  |  |  |
| TSH [μIU/ml] | | 0.353 | -0.203 | 0.91 | 0.213 |  |  |  |  |
| fT3 [pmol/l] | | -0.57 | -1.511 | 0.371 | 0.235 |  |  |  |  |
| fT4 [pmol/l] | | -0.018 | -0.407 | 0.37 | 0.927 |  |  |  |  |
| anti-TPO [IU/ml] | | 0.005 | -0.014 | 0.024 | 0.634 |  |  |  |  |
| anti-TG [IU/ml] | | 0 | -0.007 | 0.007 | 0.99 |  |  |  |  |
| Fasting glucose [mmol/l] | | 0.516 | -1.952 | 2.984 | 0.682 |  |  |  |  |
| 120' 75OGTT glucose [mmol/l] | | -0.035 | -1.094 | 1.025 | 0.949 |  |  |  |  |
| Fasting insulin [μU/ml] | | -0.154 | -0.316 | 0.009 | 0.064 | -0.694 | -1.43 | 0.042 | 0.065 |
| 120' 75OGTT insulin [μU/ml] | | -0.011 | -0.033 | 0.011 | 0.326 |  |  |  |  |
| HOMA-IR [n] | | -0.423 | -1.063 | 0.218 | 0.195 | 2.535 | -0.321 | 5.391 | 0.082 |
| Total cholesterol [mmol/l] | | 1.607 | -0.09 | 3.305 | 0.063 | -2.02 | -9.347 | 5.306 | 0.588 |
| HDL cholesterol [mmol/l] | | 3.991 | 0.52 | 7.463 | 0.024 * | 2.865 | -4.29 | 10.019 | 0.432 |
| LDL cholesterol [mmol/l] | | 1.3 | -0.672 | 3.271 | 0.196 | 3.031 | -4.922 | 10.983 | 0.455 |
| Triglycerides [mmol/l] | | -1.546 | -4.449 | 1.358 | 0.296 |  |  |  |  |
| CRP [mg/l] | | -0.187 | -0.593 | 0.22 | 0.367 |  |  |  |  |

95%CI - 95% confidence interval, * statistically significant (p<0.05), mFG - modified Ferriman-Gallwey scale score, LS - Ludwig scale score, BMI - body mass index, FSH - follicle-stimulating hormone, LH - luteinizing hormone, SHBG - sex hormone binding globulin, FAI - free androgen index, DHEA-S - dehydroepiandrosterone sulphate, TSH - thyroid-stimulating hormone, fT3 - free triiodothyronine, fT4 - free thyroxine, TPOAb - thyroid peroxidase antibodies, TGAb - thyroglobulin antibodies, 75OGTT - 75-g oral glucose tolerance test, HOMA-IR - homeostasis model assessment for insulin resistance, HDL - high-density lipoprotein, LDL - low-density lipoprotein, CRP - C-reactive protein

Supplementary Table 9. Impact of selected variables on Health-Related Quality of Life (HRQoL) in General Health domain among women with irregular menstruation.

| **Variable** | | **Univariate models** | | | | **Multivariate model** | | | |
| --- | --- | --- | --- | --- | --- | --- | --- | --- | --- |
|  |  | **Parameter** | **95%CI** | | **p** | **Parameter** | **95%CI** | | **p** |
| Age [years] | | -0.17 | -0.311 | -0.028 | 0.019 * | -0.073 | -0.232 | 0.086 | 0.366 |
| mFG | | -0.406 | -0.571 | -0.241 | <0.001 * | -0.279 | -0.454 | -0.105 | 0.002 * |
| LS | 0, I-1 | ref. |  |  |  | ref. |  |  |  |
|  | I-2 | -2.898 | -4.673 | -1.124 | 0.001 * | -2.34 | -4.119 | -0.561 | 0.01 * |
|  | I-3 | -7.357 | -10.297 | -4.418 | <0.001 * | -5.865 | -8.844 | -2.886 | <0.001 * |
|  | I-4, II-1, II-2 | -4.403 | -10.387 | 1.581 | 0.149 | -2.205 | -8.193 | 3.783 | 0.47 |
| BMI [kg/m²] | | -0.341 | -0.465 | -0.216 | <0.001 * | -0.137 | -0.338 | 0.064 | 0.181 |
| Cycle length [days] | | 0.003 | -0.006 | 0.013 | 0.522 |  |  |  |  |
| Infertility | No | ref. |  |  |  | ref. |  |  |  |
|  | Yes | -1.64 | -4.146 | 0.865 | 0.199 | 0.142 | -2.473 | 2.756 | 0.915 |
| Dysmenorrhea | No | ref. |  |  |  | ref. |  |  |  |
|  | Yes | -1.627 | -3.196 | -0.058 | 0.042 * | -0.759 | -2.483 | 0.965 | 0.388 |
| Abnormal uterine  bleeding | No | ref. |  |  |  | ref. |  |  |  |
|  | HMB | -3.201 | -4.932 | -1.469 | <0.001 * | -2.349 | -4.255 | -0.443 | 0.016 * |
|  | IMB | -2.58 | -5.545 | 0.386 | 0.088 | -1.802 | -4.779 | 1.174 | 0.235 |
| Right Ovarian Volume [ml] | | -0.003 | -0.113 | 0.108 | 0.96 |  |  |  |  |
| Left Ovarian Volume [ml] | | 0.018 | -0.057 | 0.093 | 0.638 |  |  |  |  |
| Polycystic ovarian morphology | No | ref. |  |  |  |  |  |  |  |
|  | Yes | -0.769 | -2.349 | 0.811 | 0.34 |  |  |  |  |
| Dominant follicle | No | ref. |  |  |  |  |  |  |  |
|  | Yes | -0.041 | -2.173 | 2.092 | 0.97 |  |  |  |  |
| Corpus Luteum | No | ref. |  |  |  |  |  |  |  |
|  | Yes | 0.878 | -1.762 | 3.518 | 0.514 |  |  |  |  |
| Simple Cyst | No | ref. |  |  |  |  |  |  |  |
|  | Yes | 0.655 | -3.379 | 4.69 | 0.75 |  |  |  |  |
| Ovarian lesion | No | ref. |  |  |  |  |  |  |  |
|  | Yes | -1.695 | -4.818 | 1.427 | 0.287 |  |  |  |  |
| Anti-Müllerian hormone [pmol/l] | | -0.003 | -0.027 | 0.02 | 0.782 |  |  |  |  |
| FSH [mIU/ml] | | -0.024 | -0.123 | 0.075 | 0.641 |  |  |  |  |
| LH [mIU/ml] | | 0.05 | -0.019 | 0.119 | 0.158 | 0.035 | -0.033 | 0.103 | 0.308 |
| Estradiol [pmol/l] | | 0 | -0.002 | 0.002 | 0.978 |  |  |  |  |
| Prolactin [μIU/ml] | | 0.002 | 0 | 0.005 | 0.106 | 0.002 | 0 | 0.005 | 0.087 |
| Testosterone [nmol/l] | | -0.589 | -1.539 | 0.36 | 0.224 |  |  |  |  |
| SHBG [nmol/l] | | 0.027 | 0.002 | 0.052 | 0.036 * | -0.013 | -0.043 | 0.018 | 0.418 |
| FAI [n] | | -0.109 | -0.238 | 0.02 | 0.099 | 0.02 | -0.127 | 0.167 | 0.788 |
| DHEA-S [μmol/l] | | 0.005 | -0.209 | 0.218 | 0.966 |  |  |  |  |
| TSH [μIU/ml] | | 0.151 | -0.151 | 0.454 | 0.325 |  |  |  |  |
| fT3 [pmol/l] | | -0.056 | -0.567 | 0.455 | 0.83 |  |  |  |  |
| fT4 [pmol/l] | | -0.055 | -0.265 | 0.156 | 0.611 |  |  |  |  |
| anti-TPO [IU/ml] | | -0.002 | -0.013 | 0.008 | 0.665 |  |  |  |  |
| anti-TG [IU/ml] | | 0.001 | -0.003 | 0.004 | 0.706 |  |  |  |  |
| Fasting glucose [mmol/l] | | -1.293 | -2.634 | 0.048 | 0.059 | -0.23 | -2.8 | 2.341 | 0.861 |
| 120' 75OGTT glucose [mmol/l] | | -0.562 | -1.139 | 0.014 | 0.056 | 0.056 | -0.675 | 0.786 | 0.881 |
| Fasting insulin [μU/ml] | | -0.185 | -0.273 | -0.097 | <0.001 * | -0.247 | -0.88 | 0.387 | 0.445 |
| 120' 75OGTT insulin [μU/ml] | | -0.016 | -0.028 | -0.004 | 0.007 * | -0.002 | -0.021 | 0.017 | 0.848 |
| HOMA-IR [n] | | -0.719 | -1.065 | -0.373 | <0.001 * | 0.913 | -1.803 | 3.628 | 0.51 |
| Total cholesterol [mmol/l] | | -0.339 | -1.262 | 0.584 | 0.471 |  |  |  |  |
| HDL cholesterol [mmol/l] | | 3.266 | 1.387 | 5.144 | 0.001 * | 0.928 | -1.334 | 3.19 | 0.421 |
| LDL cholesterol [mmol/l] | | -0.963 | -2.033 | 0.106 | 0.077 | -0.494 | -1.638 | 0.651 | 0.398 |
| Triglycerides [mmol/l] | | -2.549 | -4.118 | -0.98 | 0.001 * | 0.167 | -1.757 | 2.091 | 0.864 |
| CRP [mg/l] | | -0.499 | -0.718 | -0.28 | <0.001 * | -0.232 | -0.484 | 0.021 | 0.072 |

95%CI - 95% confidence interval, * statistically significant (p<0.05), mFG - modified Ferriman-Gallwey scale score, LS - Ludwig scale score, BMI - body mass index, FSH - follicle-stimulating hormone, LH - luteinizing hormone, SHBG - sex hormone binding globulin, FAI - free androgen index, DHEA-S - dehydroepiandrosterone sulphate, TSH - thyroid-stimulating hormone, fT3 - free triiodothyronine, fT4 - free thyroxine, TPOAb - thyroid peroxidase antibodies, TGAb - thyroglobulin antibodies, 75OGTT - 75-g oral glucose tolerance test, HOMA-IR - homeostasis model assessment for insulin resistance, HDL - high-density lipoprotein, LDL - low-density lipoprotein, CRP - C-reactive protein

Supplementary Table 10. Impact of selected variables on Health-Related Quality of Life (HRQoL) in Health Change domain among women with irregular menstruation.

| **Variable** | | **Univariate models** | | | | **Multivariate model** | | | |
| --- | --- | --- | --- | --- | --- | --- | --- | --- | --- |
|  |  | **Parameter** | **95%CI** | | **p** | **Parameter** | **95%CI** | | **p** |
| Age [years] | | -0.045 | -0.281 | 0.19 | 0.705 |  |  |  |  |
| mFG | | -0.289 | -0.566 | -0.013 | 0.041 * | -0.104 | -0.395 | 0.188 | 0.486 |
| LS | 0, I-1 | ref. |  |  |  | ref. |  |  |  |
|  | I-2 | -3.427 | -6.386 | -0.468 | 0.023 * | -3.315 | -6.294 | -0.335 | 0.029 * |
|  | I-3 | -7.871 | -12.773 | -2.968 | 0.002 * | -7.766 | -12.714 | -2.818 | 0.002 * |
|  | I-4, II-1, II-2 | -14.956 | -24.935 | -4.977 | 0.003 * | -15.303 | -25.279 | -5.327 | 0.003 * |
| BMI [kg/m²] | | -0.277 | -0.486 | -0.068 | 0.009 * | -0.161 | -0.456 | 0.133 | 0.282 |
| Cycle length [days] | | 0.021 | 0.005 | 0.037 | 0.01 * | 0.015 | -0.002 | 0.031 | 0.089 |
| Infertility | No | ref. |  |  |  |  |  |  |  |
|  | Yes | -0.214 | -4.374 | 3.945 | 0.919 |  |  |  |  |
| Dysmenorrhea | No | ref. |  |  |  | ref. |  |  |  |
|  | Yes | -2.762 | -5.364 | -0.16 | 0.038 * | -1.42 | -4.128 | 1.287 | 0.304 |
| Abnormal uterine  bleeding | No | ref. |  |  |  |  |  |  |  |
|  | HMB | -0.995 | -3.885 | 1.896 | 0.5 |  |  |  |  |
|  | IMB | -2.683 | -7.634 | 2.268 | 0.288 |  |  |  |  |
| Right Ovarian Volume [ml] | | 0.038 | -0.145 | 0.221 | 0.685 |  |  |  |  |
| Left Ovarian Volume [ml] | | 0.02 | -0.104 | 0.144 | 0.749 |  |  |  |  |
| Polycystic ovarian morphology | No | ref. |  |  |  |  |  |  |  |
|  | Yes | 0.034 | -2.589 | 2.656 | 0.98 |  |  |  |  |
| Dominant follicle | No | ref. |  |  |  |  |  |  |  |
|  | Yes | -1.43 | -4.965 | 2.106 | 0.428 |  |  |  |  |
| Corpus Luteum | No | ref. |  |  |  | ref. |  |  |  |
|  | Yes | 3.92 | -0.453 | 8.292 | 0.079 | 4.744 | 0.369 | 9.12 | 0.034 * |
| Simple Cyst | No | ref. |  |  |  | ref. |  |  |  |
|  | Yes | 5.813 | -0.869 | 12.496 | 0.088 | 6.274 | -0.312 | 12.861 | 0.062 |
| Ovarian lesion | No | ref. |  |  |  |  |  |  |  |
|  | Yes | 1.639 | -3.543 | 6.821 | 0.535 |  |  |  |  |
| Anti-Müllerian hormone [pmol/l] | | 0.021 | -0.018 | 0.06 | 0.287 |  |  |  |  |
| FSH [mIU/ml] | | 0.017 | -0.147 | 0.182 | 0.837 |  |  |  |  |
| LH [mIU/ml] | | 0.046 | -0.069 | 0.16 | 0.433 |  |  |  |  |
| Estradiol [pmol/l] | | 0 | -0.004 | 0.003 | 0.962 |  |  |  |  |
| Prolactin [μIU/ml] | | -0.001 | -0.005 | 0.004 | 0.731 |  |  |  |  |
| Testosterone [nmol/l] | | -1.2 | -2.774 | 0.375 | 0.135 | -0.314 | -2.542 | 1.913 | 0.782 |
| SHBG [nmol/l] | | 0.035 | -0.006 | 0.077 | 0.096 | -0.007 | -0.058 | 0.043 | 0.777 |
| FAI [n] | | -0.202 | -0.416 | 0.012 | 0.065 | -0.095 | -0.433 | 0.243 | 0.581 |
| DHEA-S [μmol/l] | | -0.108 | -0.462 | 0.246 | 0.549 |  |  |  |  |
| TSH [μIU/ml] | | -0.501 | -1.001 | 0 | 0.05 * | -0.43 | -0.928 | 0.068 | 0.091 |
| fT3 [pmol/l] | | -0.516 | -1.363 | 0.331 | 0.232 |  |  |  |  |
| fT4 [pmol/l] | | 0.197 | -0.152 | 0.547 | 0.268 |  |  |  |  |
| anti-TPO [IU/ml] | | 0.01 | -0.007 | 0.027 | 0.251 |  |  |  |  |
| anti-TG [IU/ml] | | -0.001 | -0.007 | 0.005 | 0.742 |  |  |  |  |
| Fasting glucose [mmol/l] | | -0.839 | -3.068 | 1.391 | 0.461 |  |  |  |  |
| 120' 75OGTT glucose [mmol/l] | | 0.375 | -0.585 | 1.334 | 0.444 |  |  |  |  |
| Fasting insulin [μU/ml] | | -0.061 | -0.208 | 0.085 | 0.411 |  |  |  |  |
| 120' 75OGTT insulin [μU/ml] | | -0.006 | -0.026 | 0.014 | 0.528 |  |  |  |  |
| HOMA-IR [n] | | -0.178 | -0.757 | 0.401 | 0.547 |  |  |  |  |
| Total cholesterol [mmol/l] | | 1.105 | -0.424 | 2.635 | 0.157 | -29.194 | -52.621 | -5.766 | 0.015 * |
| HDL cholesterol [mmol/l] | | 2.722 | -0.408 | 5.851 | 0.088 | 28.662 | 5.498 | 51.826 | 0.015 * |
| LDL cholesterol [mmol/l] | | 1.265 | -0.51 | 3.04 | 0.162 | 30.48 | 7.314 | 53.647 | 0.01 * |
| Triglycerides [mmol/l] | | -2.267 | -4.879 | 0.345 | 0.089 | 11.851 | 1.017 | 22.685 | 0.032 * |
| CRP [mg/l] | | -0.245 | -0.611 | 0.12 | 0.188 | 0.02 | -0.396 | 0.436 | 0.926 |

95%CI - 95% confidence interval, * statistically significant (p<0.05), mFG - modified Ferriman-Gallwey scale score, LS - Ludwig scale score, BMI - body mass index, FSH - follicle-stimulating hormone, LH - luteinizing hormone, SHBG - sex hormone binding globulin, FAI - free androgen index, DHEA-S - dehydroepiandrosterone sulphate, TSH - thyroid-stimulating hormone, fT3 - free triiodothyronine, fT4 - free thyroxine, TPOAb - thyroid peroxidase antibodies, TGAb - thyroglobulin antibodies, 75OGTT - 75-g oral glucose tolerance test, HOMA-IR - homeostasis model assessment for insulin resistance, HDL - high-density lipoprotein, LDL - low-density lipoprotein, CRP - C-reactive protein

Supplementary Table 11. Impact of selected variables on the Health-Related Quality of Life (HRQoL) domains in women with regular menstruation.

| **Physical**  **Functioning** | **Variable** | | **Univariate models** | | | | **Multivariate model** | | | |
| --- | --- | --- | --- | --- | --- | --- | --- | --- | --- | --- |
|  |  |  | **Parameter** | **95%CI** | | **p** | **Parameter** | **95%CI** | | **p** |
|  | Age [years] | | -0.101 | -0.318 | 0.115 | 0.358 |  |  |  |  |
|  | mFG [n] | | -0.27 | -0.588 | 0.047 | 0.095 | -0.164 | -0.479 | 0.151 | 0.306 |
|  | LS | 0, I-1 | ref. |  |  |  | ref. |  |  |  |
|  |  | I-2 | -2.344 | -5.253 | 0.566 | 0.114 | -2.371 | -5.266 | 0.525 | 0.108 |
|  |  | I-3 | -5.787 | -11.206 | -0.368 | 0.036 * | -5.168 | -10.54 | 0.204 | 0.059 |
|  |  | I-4, II-1, II-2 | -11.502 | -19.559 | -3.444 | 0.005 * | -10.332 | -18.319 | -2.345 | 0.011 * |
|  | BMI [kg/m²] | | -0.402 | -0.643 | -0.162 | 0.001 * | -0.36 | -0.6 | -0.119 | 0.004 * |
| **Role Limitations**  **due to Physical Health** | Age [years] | | 0.629 | 0.001 | 1.257 | 0.05 * | 0.541 | -0.113 | 1.196 | 0.105 |
|  | mFG [n] | | -0.825 | -1.751 | 0.101 | 0.081 | -0.697 | -1.638 | 0.243 | 0.146 |
|  | LS | 0, I-1 | ref. |  |  |  | ref. |  |  |  |
|  |  | I-2 | -8.246 | -16.807 | 0.314 | 0.059 | -6.567 | -15.142 | 2.009 | 0.133 |
|  |  | I-3 | -11.327 | -27.269 | 4.615 | 0.163 | -12.9 | -28.945 | 3.145 | 0.115 |
|  |  | I-4, II-1, II-2 | -18.47 | -42.174 | 5.235 | 0.126 | -21.415 | -45.087 | 2.257 | 0.076 |
|  | BMI [kg/m²] | | 0.581 | -0.128 | 1.289 | 0.108 | 0.573 | -0.154 | 1.3 | 0.122 |
| **Role Limitations**  **due to Emotional Problems** | Age [years] | | 1.08 | 0.404 | 1.756 | 0.002 * | 0.997 | 0.316 | 1.677 | 0.004 * |
|  | mFG [n] | | -1.518 | -2.514 | -0.522 | 0.003 * | -1.238 | -2.227 | -0.249 | 0.014 * |
|  | LS | 0, I-1 | ref. |  |  |  | ref. |  |  |  |
|  |  | I-2 | -11.268 | -20.485 | -2.052 | 0.017 * | -8.797 | -17.904 | 0.311 | 0.058 |
|  |  | I-3 | 1.34 | -15.824 | 18.503 | 0.878 | -0.473 | -17.53 | 16.583 | 0.957 |
|  |  | I-4, II-1, II-2 | -34.108 | -59.63 | -8.586 | 0.009 * | -36.489 | -61.599 | -11.38 | 0.005 * |
|  | BMI [kg/m²] | | 0.444 | -0.327 | 1.214 | 0.258 |  |  |  |  |
| **Energy/Fatigue** | Age [years] | | 0.463 | 0.149 | 0.777 | 0.004 * | 0.427 | 0.108 | 0.747 | 0.009 * |
|  | mFG [n] | | -0.704 | -1.166 | -0.241 | 0.003 * | -0.615 | -1.08 | -0.151 | 0.01 * |
|  | LS | 0, I-1 | ref. |  |  |  | ref. |  |  |  |
|  |  | I-2 | -2.494 | -6.818 | 1.83 | 0.257 | -1.348 | -5.626 | 2.929 | 0.536 |
|  |  | I-3 | 1.465 | -6.588 | 9.518 | 0.721 | 0.806 | -7.205 | 8.817 | 0.843 |
|  |  | I-4, II-1, II-2 | -10.598 | -22.573 | 1.376 | 0.083 | -11.556 | -23.35 | 0.237 | 0.055 |
|  | BMI [kg/m²] | | -0.082 | -0.44 | 0.276 | 0.654 |  |  |  |  |
| **Emotional Well-Being** | Age [years] | | 0.333 | 0.022 | 0.644 | 0.036 * | 0.353 | 0.035 | 0.672 | 0.03 * |
|  | mFG [n] | | -0.766 | -1.219 | -0.313 | 0.001 * | -0.623 | -1.081 | -0.165 | 0.008 * |
|  | LS | 0, I-1 | ref. |  |  |  | ref. |  |  |  |
|  |  | I-2 | -3.811 | -8.023 | 0.401 | 0.076 | -2.89 | -7.064 | 1.285 | 0.174 |
|  |  | I-3 | -0.618 | -8.462 | 7.226 | 0.877 | -0.717 | -8.527 | 7.093 | 0.857 |
|  |  | I-4, II-1, II-2 | -17.126 | -28.789 | -5.463 | 0.004 * | -17.064 | -28.587 | -5.542 | 0.004 * |
|  | BMI [kg/m²] | | -0.272 | -0.623 | 0.079 | 0.129 | -0.264 | -0.618 | 0.09 | 0.143 |
| **Social Functioning** | Age [years] | | 0.293 | -0.145 | 0.732 | 0.189 | 0.217 | -0.226 | 0.66 | 0.336 |
|  | mFG [n] | | -1.037 | -1.674 | -0.4 | 0.002 * | -0.946 | -1.59 | -0.302 | 0.004 * |
|  | LS | 0, I-1 | ref. |  |  |  | ref. |  |  |  |
|  |  | I-2 | -5.794 | -11.74 | 0.152 | 0.056 | -4.562 | -10.491 | 1.367 | 0.131 |
|  |  | I-3 | 0.166 | -10.907 | 11.239 | 0.976 | 0.71 | -10.393 | 11.813 | 0.9 |
|  |  | I-4, II-1, II-2 | -17.294 | -33.759 | -0.829 | 0.04 * | -17.314 | -33.66 | -0.968 | 0.038 * |
|  | BMI [kg/m²] | | 0.025 | -0.47 | 0.519 | 0.922 |  |  |  |  |
| **Pain** | Age [years] | | 0.29 | -0.095 | 0.676 | 0.14 | 0.293 | -0.101 | 0.687 | 0.145 |
|  | mFG [n] | | -0.234 | -0.803 | 0.336 | 0.42 |  |  |  |  |
|  | LS | 0, I-1 | ref. |  |  |  | ref. |  |  |  |
|  |  | I-2 | -4.24 | -9.512 | 1.032 | 0.115 | -3.887 | -9.171 | 1.398 | 0.149 |
|  |  | I-3 | -3.454 | -13.272 | 6.364 | 0.489 | -4.491 | -14.391 | 5.409 | 0.373 |
|  |  | I-4, II-1, II-2 | -3.375 | -17.974 | 11.224 | 0.65 | -4.342 | -18.974 | 10.29 | 0.56 |
|  | BMI [kg/m²] | | -0.144 | -0.58 | 0.291 | 0.515 |  |  |  |  |
| **General Health** | Age [years] | | -0.048 | -0.265 | 0.17 | 0.667 |  |  |  |  |
|  | mFG [n] | | -0.58 | -0.894 | -0.266 | <0.001 * | -0.474 | -0.789 | -0.16 | 0.003 * |
|  | LS | 0, I-1 | ref. |  |  |  | ref. |  |  |  |
|  |  | I-2 | -3.611 | -6.541 | -0.681 | 0.016 * | -3.291 | -6.179 | -0.404 | 0.026 * |
|  |  | I-3 | -6.748 | -12.204 | -1.292 | 0.016 * | -5.756 | -11.112 | -0.399 | 0.035 * |
|  |  | I-4, II-1, II-2 | -4.367 | -12.48 | 3.746 | 0.29 | -3.123 | -11.087 | 4.842 | 0.441 |
|  | BMI [kg/m²] | | -0.355 | -0.597 | -0.113 | 0.004 * | -0.307 | -0.547 | -0.067 | 0.012 * |
| **Health Change** | Age [years] | | -0.032 | -0.392 | 0.328 | 0.861 |  |  |  |  |
|  | mFG [n] | | -0.005 | -0.535 | 0.524 | 0.984 |  |  |  |  |
|  | LS | 0, I-1 | ref. |  |  |  | ref. |  |  |  |
|  |  | I-2 | -7.098 | -11.96 | -2.236 | 0.004 * | -7.098 | -11.96 | -2.236 | 0.004 * |
|  |  | I-3 | -1.886 | -10.941 | 7.168 | 0.682 | -1.886 | -10.941 | 7.168 | 0.682 |
|  |  | I-4, II-1, II-2 | -3.077 | -16.541 | 10.387 | 0.653 | -3.077 | -16.541 | 10.387 | 0.653 |
|  | BMI [kg/m²] | | -0.249 | -0.653 | 0.155 | 0.226 |  |  |  |  |

95%CI - 95% confidence interval, * statistically significant (p<0.05), mFG - modified Ferriman-Gallwey scale score, LS - Ludwig scale score, BMI - body mass index
